# A novel explainable online calculator for contrast-induced AKI in diabetics: a multi-centre validation and prospective evaluation study

**DOI:** 10.1186/s12967-023-04387-x

**Published:** 2023-07-31

**Authors:** Mengqing Ma, Xin Wan, Yuyang Chen, Zhichao Lu, Danning Guo, Huiping Kong, Binbin Pan, Hao Zhang, Dawei Chen, Dongxu Xu, Dong Sun, Hong Lang, Changgao Zhou, Tao Li, Changchun Cao

**Affiliations:** 1 Department of Nephrology, Sir Run Run Hospital, Nanjing Medical University, Nanjing, 211166 Jiangsu China; 2 Department of Nephrology, Nanjing First Hospital, Nanjing Medical University, Nanjing, 210006 Jiangsu China; 3 Department of Computer Science and Technology, Nanjing University, Nanjing, 210023 Jiangsu China; 4 Department of Cardiology, Sir Run Run Hospital, Nanjing Medical University, Nanjing, 211166 Jiangsu China; 5 Department of Nephrology, Affiliated Hospital of Xuzhou Medical University, Xuzhou, 221000 Jiangsu China; 6 Department of Cardiology, Affiliated Shu Yang Hospital of Nanjing University of Chinese Medicine, Shuyang, 223600 Jiangsu China

**Keywords:** Contrast-induced acute kidney injury, Machine learning, Web calculator, Predictive models, Diabetes mellitus

## Abstract

**Background:**

In patients undergoing percutaneous coronary intervention (PCI), contrast-induced acute kidney injury (CIAKI) is a frequent complication, especially in diabetics, and is connected with severe mortality and morbidity in the short and long term. Therefore, we aimed to develop a CIAKI predictive model for diabetic patients.

**Methods:**

3514 patients with diabetes from four hospitals were separated into three cohorts: training, internal validation, and external validation. We developed six machine learning (ML) algorithms models: random forest (RF), gradient-boosted decision trees (GBDT), logistic regression (LR), least absolute shrinkage and selection operator with LR, extreme gradient boosting trees (XGBT), and support vector machine (SVM). The area under the receiver operating characteristic curve (AUC) of ML models was compared to the prior score model, and developed a brief CIAKI prediction model for diabetes (BCPMD). We also validated BCPMD model on the prospective cohort of 172 patients from one of the hospitals. To explain the prediction model, the shapley additive explanations (SHAP) approach was used.

**Results:**

In the six ML models, XGBT performed best in the cohort of internal (AUC: 0.816 (95% CI 0.777–0.853)) and external validation (AUC: 0.816 (95% CI 0.770–0.861)), and we determined the top 15 important predictors in XGBT model as BCPMD model variables. The features of BCPMD included acute coronary syndromes (ACS), urine protein level, diuretics, left ventricular ejection fraction (LVEF) (%), hemoglobin (g/L), congestive heart failure (CHF), stable Angina, uric acid (umol/L), preoperative diastolic blood pressure (DBP) (mmHg), contrast volumes (mL), albumin (g/L), baseline creatinine (umol/L), vessels of coronary artery disease, glucose (mmol/L) and diabetes history (yrs). Then, we validated BCPMD in the cohort of internal validation (AUC: 0.819 (95% CI 0.783–0.855)), the cohort of external validation (AUC: 0.805 (95% CI 0.755–0.850)) and the cohort of prospective validation (AUC: 0.801 (95% CI 0.688–0.887)). SHAP was constructed to provide personalized interpretation for each patient. Our model also has been developed into an online web risk calculator. MissForest was used to handle the missing values of the calculator.

**Conclusion:**

We developed a novel risk calculator for CIAKI in diabetes based on the ML model, which can help clinicians achieve real-time prediction and explainable clinical decisions.

**Supplementary Information:**

The online version contains supplementary material available at 10.1186/s12967-023-04387-x.

## Introduction

As radiography and percutaneous coronary intervention (PCI) have grown more widely used, contrast-induced acute kidney injury (CIAKI) has risen to become the third most prevalent cause of iatrogenic acute kidney injury [1], especially in patients with diabetes mellitus (DM) due to the poor vascular conditions [2, 3]. As many as 21.2% of DM patients may suffer from CIAKI [4], which may lead to as high as 30% mortality from CIAKI [5]. Therefore, an early predictive system applied in diabetic patients according to their risk of CIAKI is crucial to reduce the frequency of CIAKI.

Serum creatinine levels are still used in the current definition of CIAKI, which could delay the diagnosis of CIAKI. Although some novel biomarkers have been proven to predict CIAKI [6, 7], cost-effectiveness limits their widely applications [8]. Clinical risk scores like the Mehran score [9] have been introduced into clinical practice for decades. However, the predictive power was inadequate in different races or populations. Recently, several studies have demonstrated that the machine learning (ML) model has an excellent prediction performance in kidney disease compared with the traditional statistics model [10–13]. ML model has a more accurate prediction ability because it gives the probability of events individually rather than risk groups.

However, these ML models rarely explained the models’ variables because of the shortcomings of the black box in ML algorithms. Most studies often lacked the verification of external data sets. Furthermore, there are few prediction models based on the website for clinical use. We intended to apply a range of ML algorithms to establish ML models and compare the models’ prediction performance to the Mehran score [9]. In addition, we used data from multi-centre hospitals as an external cohort and one of the centers as a prospective cohort to validate our model. Then, we established a dynamic and explainable website tool for predicting CIAKI in patients with diabetes.

## Methods

### Study design and participants

The study was divided into two steps. Firstly, we retrospectively reviewed the medical records from multi-center hospitals to build and validate the predictive model. The multi-centre hospitals included Affiliated Sir Run Run Hospital of Nanjing Medical University, Nanjing First Hospital, Affiliated Shu Yang Hospital of Nanjing University of Chinese Medicine and Xu Zhou Medical University Hospital. The study population included diabetic patients who underwent coronary angiography (CAG) and PCI between January 2014 and January 2020. We excluded patients based on the following criteria: (1) missing serum creatinine levels prior to and after CAG and PCI; (2) needing dialysis before CAG and PCI; (3) repeated hospitalization for PCI; and (4) acute kidney injury prior to CAG and PCI. Our research was carried out in respect to the Declaration of Helsinki. Due to its retrospective design, our hospitals gave their approval for the study and waived the need for informed consent.

Secondly, we conducted a prospective study in Affiliated Sir Run Run Hospital of Nanjing Medical University to determine early prediction of CIAKI with our CIAKI online calculator. The study population included adult diabetic patients that underwent CAG and PCI from June 2021 to April 2022. The Ethnic Committee approved this study (Ethics number: 2021-SR-041) and waived the requirement for informed permission to use identifiable data. We reported our work following the Transparent Reporting of a multivariable prediction model for Individual Prognosis Or Diagnosis (TRIPOD) statement guideline [14], Strengthening the Reporting of Observational Studies in Epidemiology (STROBE) statement [15] and guidelines for ML predictive modeling [16].

### Clinical endpoints

CIAKI was the primary outcome of our study, based on the Contrast Media Safety Committee (CMSC), described as an increment of serum creatinine value at least 44.2 μmol/L (0.5 mg/dl) or 1.25 times comparing the baseline level within 72 h exposure to contrast agent, eliminating alternative causes of acute kidney injury. The baseline creatinine was the lowest serum creatinine level within 7 days before CAG. In the 72 h following CAG and PCI, all serum creatinine values were collected. Dialysis, stroke, length of in-hospital stay, the new-onset or recurrence of myocardial infarction and other adverse cardiovascular events such as worsening heart failure and death were also included as outcomes.

### Other definitions

DM was defined if the patient’s treatment included dietary, oral, or insulin therapy or if patients’ fasting blood glucose value was 126 mg/dl based on the practice guidelines of the American Diabetes Association [17]. Congestive heart failure (CHF) was diagnosed if the patients were grouped into New York Heart Association (NYHA) class III or higher based on the categorization system of the NYHA or history of pulmonary edema. Clinicians comprehensively diagnosed acute coronary syndromes (ACS) according to the symptoms of myocardial ischemia, changes in electrocardiogram, and myocardial injury biomarkers [18]. According to the definition of chronic kidney disease (CKD), patients with proteinuria, estimated glomerular filtration rate (eGFR) < 60 ml/min/1.73m^2^, or both on at least two occasions more than or equal to three months apart [19, 20].

### Data collection and preprocessing

In each institution, demographic data, preoperative medications, and laboratory tests were collected, including gender, age, pre-CAG blood pressure, body mass index (BMI), coronary artery disease, primary disease, contrast agents, and periprocedural biochemical markers. We removed characteristics absent in 11% or more of the samples. The abnormal value of variables were rechecked in electronic hospital records. Otherwise, they were treated as missing values. Categorical variables were processed with one-hot encoding and label encoding. One-hot encoding creates a separate binary feature for each category and is suitable for categorical variables without a specific order or hierarchy. For example, we converted the gender “male” or “female” to “female or not”. Label encoding assigns a unique numerical label to each category. Each category is mapped to a different integer value. Label encoding is suitable for categorical variables with a clear order or hierarchy, such as ordinal variables. For example, the variable “Diabetes history (yrs)” with categories “ < 1 year, 1–5 years, 5–10 years, 10–20 years, >  = 20 years” were converted to “1, 2, 3, 4, 5”. Variance inflation factor (VIF) and generalized variance inflation factor (GVIF) were used to deal with collinearity between continuous and categorical variables, respectively. The continuous variables with VIF > 10 were removed. For categorical variables, we set the category with the largest proportion in each categorical variable as the reference level and considered the categorical variables with GVIF^[1/(2 × Df)] > 10^(1/2) to have high collinearity and removed them, where Df refers to the degree of freedom. We divided the data into the cohort of training, internal validation and external validation. We randomly used 80% of the data from Nanjing First Hospital for model training, 20% from Nanjing First Hospital for model internal validation, and other centres for model external validation. We used the missForest method, which can handle missing values with a combination of continuous and categorical variables to fill each remaining measurement’s missing data in the three cohorts separately [21]. Meanwhile, variables were standardized before training and prediction by removing the mean and scaling to unit variance.

In the prospective design, we recorded the time of each variable in the CIAKI model and the time of clinical diagnosis of CIAKI to obtain the earliest time when the model predicted the occurrence of CIAKI. Because of the prospective design, none of the required variables had a missing value.

### Data balancing

To solve the imbalance between positive and negative samples, we adopt a variety of balancing methods in the training set, including oversampling and undersampling. Oversampling includes Synthetic Minority Oversampling Technique (SMOTE), ADAptive SYNthetic (ADASYN) technique, and random oversampling. Undersampling includes random undersampling and TomekLinks (Additional file 1: Table S4). Finally, we found that each balancing method performed equally on the internal validation set, but TomekLinks performed better in the external validation set, so we chose to use TomekLinks. Specifically, TomekLinks focuses on neighboring pairs of samples, where one sample belongs to the minority class and the other belongs to the majority class. These sample pairs are close to each other and form links. These links are considered potential noise or outliers, which may have a negative impact on model training and performance. By identifying and addressing these links, we can reduce noise or outliers in the data and improve the performance of the classification model.

### Mehran risk score

Mehran risk score [9] is calculated with 8 variables: hypotension, CHF, intra-arterial balloon pump (IABP), anemia, age, diabetes, contrast media volume, serum creatinine or eGFR. We calculated the total Mehran risk scores for each patient based on the sum of the scores corresponding to the 8 variables which were 5 points for hypotension (Systolic blood pressure is less than 80 mmHg for at least 1 h and inotropic assistance is required), 5 points for IABP (IABP is used), 5 points for CHF (NYHA classification III/IV or history of pulmonary edema), 4 points for age (more than 75 years old), 3 points for anemia (men’s hematocrit less than 39% while women’s less than 36%.), 3 points for diabetes, 1 point for contrast media volume per 100ml and 4 points for serum creatinine > 1.5mg/dl, or 2 points for eGFR 40–60 ml/min/1.73m^2^, 4 points for eGFR 20–40 ml/min/1.73m^2^, and 6 points for eGFR < 20 ml/min/1.73m^2^.

### Machine learning development

Six ML models were constructed, including extreme gradient boosting trees (XGBT) model, random forest (RF) model, support vector machine (SVM) model, logistic regression (LR) model, least absolute shrinkage and selection operator (LASSO) with LR (Lasso + LR), and gradient boosted decision trees (GBDT) model. Additional file 1 included a full explanation of the six ML models.

ML models were also trained using ten-fold cross-validation (Additional file 1**: **Figure S1 and Additional file 1: Table S3). The initial samples were randomly split into ten equal-sized subsamples, one of which was used to validate the results and the other nine as training samples. For each model, in order to select the ideal hyperparameters, a grid search method with ten-fold cross validation was used. Furthermore, we constructed the SHapley Additive exPlanation (SHAP), which demonstrates each variable’s impact on the overall model as well as its contributions to the model. Additionally, the SHAP plot function was also used to reveal the XGBT model’s complicated link between factors and results. Finally, to forecast the risk of CIAKI in diabetics, we developed an explainable online web-based risk calculator.

### Performance evaluation

All models were evaluated in internal as well as external validation sets. Each model’s area under the receiver operating characteristic curve (AUC), accuracy, positive predictive value (PPV), negative predictive value (NPV), sensitivity, specificity, and *F*_*1*_ score were also compared. Additionally, we chose the CIAKI prediction threshold by maximizing the *F*_*1*_ score in the training set. A 95% confidence interval (CI) was performed in 1000 iterations of bootstrap sampling with replacement. To examine the agreement between calculated likelihood and observed CIAKI prevalence in the population, a calibration curve was utilized. Moreover, the net benefit of each model was calculated using decision curve analysis (DCA) based on the difference between the predicted benefit and the expected risk associated with CIAKI.

### Statistical analysis

For descriptive analyses, categorical variables were expressed as quantities and percentages. To compare categorical variables, chi-square tests were utilized. Analysis and expression of continuous variables using the mean and standard deviation or median and interquartile range were compared using either the Independent-sample T-test or the Mann–Whitney U test. All analyses were carried out with Python version 3.9.7, R version 4.1.0, and SPSS version 22.0. *P* < 0.05 was used as the statistical significance level.

## Results

### Baseline characteristics

**Fig. 1:**
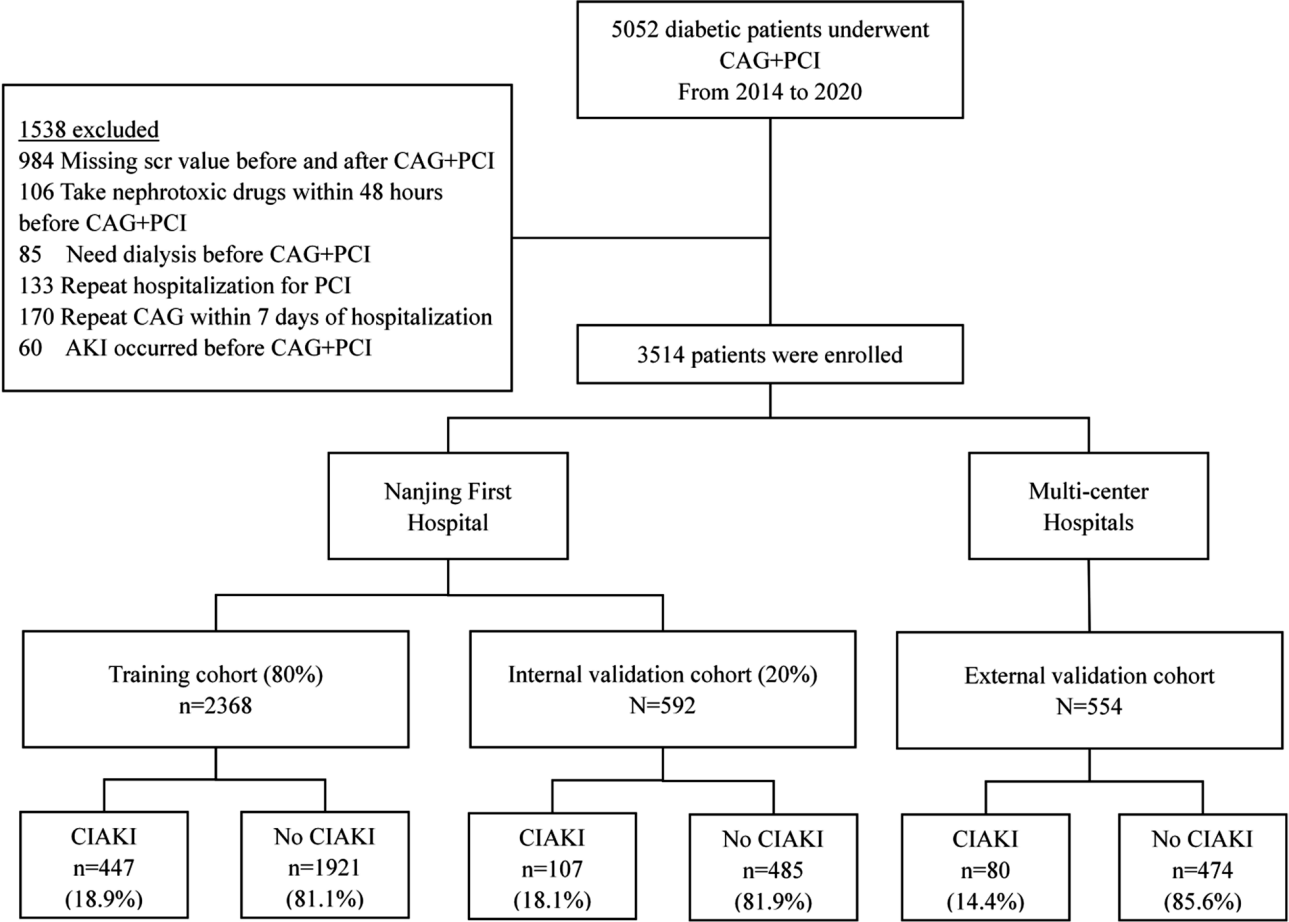
Patient enrollment process and cohort assignment

From January 1, 2014 to January 30, 2020, a total of 5052 diabetic patients underwent CAG + PCI. Based on the inclusion and exclusion criteria, 3514 patients were included in the study. The characteristics of included patients and excluded patients were compared in Additional file 1: Table S1. Patients that were excluded had more CKD stage 4–5, higher blood urea nitrogen levels and more vessels of coronary artery disease. The main reason was that the exclusion criteria included patients who were on dialysis and had developed acute kidney injury before CAG + PCI which resulted in excluded patients having worse kidney function. Moreover, the exclusion criteria included repeated hospitalization and repeated CAG which suggested that the excluded patient’s coronary artery disease might be more serious like more vessels of coronary artery disease and required multiple CAG examinations. The remaining characteristics were not statistically different between the included and excluded patients. We randomly partitioned patients from Nanjing First Hospital into the cohort of training (80%, 2368 patients) and the cohort of internal validation (20%, 592 patients). Patients from the other three hospitals were used as the external validation cohorts (554 patients) (Fig. 1). The frequency of CIAKI in training, and internal and external validation sets was 447/2368 (18.9%), 107/592 (18.1%), and 80/554 (14.4%), respectively (Fig. 1). Additional file 1**: **Table S2 displayed the three cohorts’ initial characteristics. The median age in the cohort of training, internal validation and external validation was 67-year-old (interquartile range [IQR]: 60–74), 67-year-old (IQR: 59–73) and 65-year-old (IQR: 58–73), respectively. Patients with CIAKI were older, had worse heart dysfunction, higher frequency of CKD 3–4 stage, coronary artery disease and anemia, and higher uric acid, urine protein, and total cholesterol (Table 1).

**Table 1:** Characteristics of CIAKI and non-CIAKI patients in the three groups

Characteristics	Training set (n = 2368)			Internal validation set (n = 592)			External validation set (n = 554)		
	Non-CIAKI	CIAKI	*P*	Non-CIAKI	CIAKI	*P*	Non-CIAKI	CIAKI	*P*
Demographics									
Age, yrs	65.97 ± 10.10	68.22 ± 10.55	< 0.001	65.27 ± 10.25	68.06 ± 10.59	0.012	63.59 ± 10.95	70.65 ± 8.85	< 0.001
Female, n(%)	602 (31.3)	146 (32.7)	0.587	165 (34)	38 (35.5)	0.768	180 (38.0)	39 (48.8)	0.068
Height, cm	166.63 ± 7.70	165.99 ± 7.27	0.11	166.12 ± 7.73	166.00 ± 7.58	0.881	166.98 ± 7.73	164.34 ± 7.65	0.005
Weight, kg	70.46 ± 10.54	69.72 ± 10.74	0.183	70.07 ± 11.72	69.8 ± 11.78	0.828	70.62 ± 10.44	66.93 ± 9.01	0.003
BMI, kg/m2	25.31 ± 2.94	25.25 ± 3.23	0.693	25.33 ± 3.51	25.25 ± 3.42	0.838	25.14 ± 2.95	24.62 ± 2.87	0.146
Medical history									
Hypertension, n(%)	1467 (76.4%)	362 (81)	0.036	362 (74.6)	82 (76.6)	0.666	315 (66.5)	54 (67.5)	0.855
NYHA Classification, n(%)			< 0.001			< 0.001			< 0.001
NYHA Classification I	108 (7.8)	17 (6.3)		37 (10.7)	2 (3.1)		149 (45.3)	10 (18.9)	
NYHA Classification II	1046 (75.7)	142 (52.6)		250 (72.3)	37 (57.8)		134 (40.7)	22 (41.5)	
NYHA Classification III	201 (14.6)	94 (34.8)		49 (14.2)	19 (29.7)		38 (11.6)	17 (32.1)	
NYHA Classification IV	26 (1.9)	17 (6.3)		10 (2.9)	6 (9.4)		8 (2.4)	4 (7.5)	
CHF, n(%)	187 (10.1)	97 (22.1)	< 0.001	49 (10.5)	22 (21.4)	0.003	48 (10.4)	20 (25.0)	< 0.001
Prior myocardial infarction, n(%)	200 (10.4)	46 (10.3)	0.94	34 (7.0)	9 (8.4)	0.613	30 (6.3)	7 (8.8)	0.422
Stable Angina, n(%)	221 (11.5)	31 (6.9)	0.005	48 (9.9)	4 (3.7)	0.042	23 (4.9)	9 (11.3)	0.023
ACS, n(%)	813 (42.3)	346 (77.4)	< 0.001	198 (40.8)	82 (76.6)	< 0.001	383 (80.8)	70 (87.5)	0.151
Diabetes history, yrs			0.125			0.931			0.072
< 1	75 (4.5)	13 (3.1)		22 (5.2)	5 (5.2)		49 (10.5)	6 (7.9)	
1–5	297 (17.8)	64 (15.5)		65 (15.3)	5 (12.4)		144 (30.8)	20 (26.3)	
5–10	462 (27.7)	110 (26.6)		142 (33.4)	31 (32.0)		102 (21.8)	12 (15.8)	
10–20	649 (39.0)	164 (39.7)		146 (34.4)	37 (38.1)		124 (26.6)	22 (28.9)	
> = 20	183 (11.0)	62 (15.0)		50 (11.8)	12 (12.4)		48 (10.3)	16 (21.1)	
CKD, n(%)	211 (11.5)	115 (25.7)	< 0.001	57 (11.8)	26 (24.3)	0.001	32 (6.8)	24 (30.0)	< 0.001
Anemia, n(%)	575 (29.9)	202 (45.2)	< 0.001	143 (29.5)	48 (44.9)	0.002	81 (17.1)	25 (31.3)	0.003
CAG and PCI									
Vessels of coronary artery disease, n(%)			0.039			0.004			0.003
0	224 (11.7)	42 (9.4)		59 (12.2)	9 (8.4)		23 (4.9)	0 (0)	
1	617 (32.1)	124 (27.7)		160 (33.0)	36 (33.6)		75 (15.8)	9 (11.3)	
2	735 (38.3)	176 (39.4)		187 (38.6)	38 (35.5)		142 (30.0)	16 (20.0)	
3	281 (14.6)	79 (17.7)		64 (13.2)	12 (11.2)		190 (40.1)	38 (47.5)	
4	54 (2.8)	20 (4.5)		14 (2.9)	9 (8.4)		37 (7.8)	13 (16.3)	
5	9 (0.5)	5 (1.1)		1 (0.2)	3 (2.8)		7 (1.5)	4 (5)	
6	1 (0.1)	1 (0.2)		0 (0)	0 (0)		0 (0)	0 (0)	
Single-vessel disease, n(%)	617 (32.1)	124 (27.7)	0.072	160 (33)	36 (33.6)	0.896	75 (15.8)	9 (11.3)	0.292
Multi-vessel disease, n(%)	1080 (56.2)	281 (62.9)	0.011	266 (54.8)	62 (57.9)	0.559	376 (79.3)	71 (88.8)	0.048
Preoperative SBP, mmHg	134.11 ± 16.68	136.15 ± 20.61	0.051	133.94 ± 17.30	136.48 ± 19.95	0.183	136.35 ± 18.09	139.03 ± 21.38	0.235
Preoperative DBP, mmHg	78.73 ± 10.96	80.76 ± 12.83	0.002	78.72 ± 11.23	79.30 ± 13.44	0.641	79.21 ± 11.58	80.30 ± 12.85	0.444
Contrast agent									
Nonionic low-osmolar, n(%)	712 (37.1)	116 (26.0)	< 0.001	178 (36.7)	32 (29.9)	0.184	306 (64.6)	58 (72.5)	0.166
Nonionic iso-osmolar, n(%)	1168 (60.8)	320 (71.6)	< 0.001	292 (62.2)	71 (66.4)	0.237	185 (39.0)	24 (30.0)	0.123
Volume of contrast agent, mL	191.22 ± 68.78	203.65 ± 69.51	0.001	187.90 ± 66.72	204.91 ± 71.22	0.019	162.10 ± 90.49	167.96 ± 75.90	0.584
Medications									
Β-blocker, n(%)	1179 (31.3)	248 (55.5)	0.022	292 (60.2)	59 (55.1)	0.334	325 (68.6)	34 (42.5)	< 0.001
ACEI/ARB, n(%)	1105 (57.5)	288 (64.4)	0.008	284 (58.6)	70 (65.4)	0.19	305 (64.3)	55 (68.8)	0.445
Diuretics, n(%)	334 (17.9)	174 (39.5)	< 0.001	85 (18.1)	41 (38.3)	< 0.001	205 (43.2)	46 (57.5)	0.018
CCB, n(%)	531 (28.3)	107 (24.5)	0.111	129 (27.3)	25 (23.8)	0.461	178 (37.6)	31 (38.8)	0.838
Insulins, n(%)	952 (49.6)	259 (57.9)	0.001	227 (46.8)	60 (56.1)	0.082	148 (31.2)	31 (38.8)	0.183
Oral hypoglycemic agent, n(%)	1125 (58.6)	237 (53.0)	0.033	261 (53.8)	48 (44.9)	0.093	346 (73.0)	55 (68.8)	0.432
Pre-procedural laboratory determinations									
Glucose, mmol/L	9.25 ± 3.69	9.82 ± 3.80	0.004	9.37 ± 3.61	9.75 ± 3.08	0.318	9.17 ± 3.72	8.16 ± 1.80	< 0.001
BUN, mg/dL	6.31 ± 2.16	7.26 ± 3.62	< 0.001	6.26 ± 2.36	7.28 ± 3.37	0.004	5.99 ± 2.30	7.23 ± 2.73	< 0.001
Baseline creatinine, umol/L	76.12 ± 27.99	88.71 ± 42.36	< 0.001	76.63 ± 29.76	88.59 ± 42.32	0.006	67.42 ± 22.50	88.97 ± 40.08	< 0.001
eGFR, mL/min/1.73 m^2^	85.80 ± 19.53	76.81 ± 24.69	< 0.001	85.50 ± 20.22	76.98 ± 25.39	0.001	95.77 ± 22.50	78.82 ± 16.23	< 0.001
CKD stage, n(%)			< 0.001			0.002			< 0.001
Stage 1	976 (50.8)	160 (35.8)		240 (49.5)	40 (37.4)		314 (66.2)	19 (23.8)	
Stage 2	724 (37.7)	171 (38.3)		188 (38.8)	41 (38.3)		128 (27.0)	37 (46.3)	
Stage 3	207 (10.8)	107 (23.9)		56 (11.5)	24 (22.4)		30 (6.3)	19 (23.8)	
Stage 4	14 (0.7)	9 (2.0)		1 (0.2)	2 (1.9)		2 (0.4)	5 (6.3)	
Hemoglobin, g/L	133.05 ± 15.77	127.98 ± 19.87	< 0.001	132.68 ± 15.99	127.41 ± 19.49	0.01	135.35 ± 16.30	125.78 ± 20.55	< 0.001
Albumin, g/L	38.87 ± 3.80	37.63 ± 4.30	< 0.001	38.79 ± 3.70	38.13 ± 3.76	0.097	42.34 ± 4.24	39.27 ± 4.49	< 0.001
Uric acid, umol/L	326.18 ± 102.42	373.06 ± 133.01	< 0.001	330.11 ± 108.48	391.19 ± 125.08	< 0.001	314.63 ± 95.32	372.58 ± 105.38	< 0.001
Total cholesterol, mmol/L	3.83 ± 1.13	3.99 ± 1.18	0.01	3.83 ± 1.08	4.24 ± 1.13	< 0.001	4.28 ± 1.18	3.96 ± 0.88	0.005
Triglycerides, mmol/L	1.75 ± 1.38	1.72 ± 1.26	0.604	1.79 ± 1.19	1.90 ± 1.39	0.413	2.08 ± 1.7	1.75 ± 1.05	0.019
HDL, mmol/L	0.98 ± 0.23	0.98 ± 0.26	0.946	0.98 ± 0.23	1.03 ± 0.27	0.091	1.11 ± 0.31	1.12 ± 0.26	0.861
LDL, mmol/L	2.23 ± 0.90	2.37 ± 0.97	0.004	2.21 ± 0.89	2.46 ± 0.91	0.01	2.46 ± 0.94	2.33 ± 0.83	0.254
Urine protein level, n(%)			< 0.001			< 0.001			< 0.001
0	1722 (89.6)	353 (79)		439 (90.5)	81 (75.7)		390 (82.3)	14 (17.5)	
1	122 (6.4)	55 (12.3)		32 (6.6)	18 (16.8)		65 (13.7)	27 (33.8)	
2	52 (2.7)	31 (6.9)		11 (2.3)	5 (4.7)		17 (3.6)	28 (35.0)	
3	25 (1.3)	8 (1.8)		3 (0.6)	3 (2.8)		2 (0.4)	11 (13.8)	
Proteinuria, n(%)	199 (10.4)	94 (21.0)	< 0.001	46 (9.5)	26 (24.3)	< 0.001	77 (16.2)	64 (80.0)	< 0.001
LVEF, %	59.48 ± 9.59	55.00 ± 11.82	< 0.001	59.64 ± 9.38	54.43 ± 11.88	< 0.001	57.10 ± 7.64	51.84 ± 10.25	< 0.001

*CIAKI* contrast-induced acute kidney injury, *BMI* body mass index, *CKD* chronic kidney disease, *CHF*, congestive heart failure, *ACS* acute coronary syndrome; *SBP* systolic blood pressure, *DBP* diastolic blood pressure, *CCB* calcium channel blocker, *ACEI* angiotensin-converting enzyme inhibitor, *ARB* angiotensin receptor blocker, *eGFR* estimated glomerular filtration rate, *HDL* high-density lipoprotein, *LDL* low-density lipoprotein, *LVEF* left ventricular ejection fraction.

### Clinical endpoints

634 patients (18.04%) developed CIAKI, and 50 (1.42%) occurred adverse cardiovascular events. Of them, 21 (0.60%) patients revealed worse heart failure, and 12 (0.34%) reoccurred or experienced a new-onset myocardial infarction. On top of that, we observed 7 (0.20%) patients developed stroke, and 11 (0.31%) death. CIAKI was linked to an increased risk of myocardial infarction (0.79% vs 0.24%, *P* = 0.033) and overall adverse cardiovascular events (2.5% vs 1.2%, *P* = 0.01) and increased hospitalization stay (9.23 ± 4.87 vs 7.38 ± 4.87, *P* < 0.001) **(**Table 2**)**.

**Table 2:** Comparison of in-hospital outcomes between CIAKI and non-CIAKI patients

Outcome	CIAKI	Non-CIAKI	*P* value
Worsening heart failure (%)	0.95 (6/634)	0.52 (15/2880)	0.208
Myocardial infarction(%)	0.79 (5/634)	0.24 (7/2880)	0.033
Stroke (%)	0.16 (1/634)	0.21 (6/2880)	0.796
Deaths (%)	0.63 (4/634)	0.24 (7/2880)	0.113
Overall adverse cardiovascular events (at least 1)	2.5 (16/634)	1.2 (34/2880)	0.01
Length of in-hospital stay (d)	9.23 ± 4.87	7.38 ± 3.53	< 0.001

*CIAKI* contrast-induced acute kidney injury

### Feature selection

**Fig. 2:**
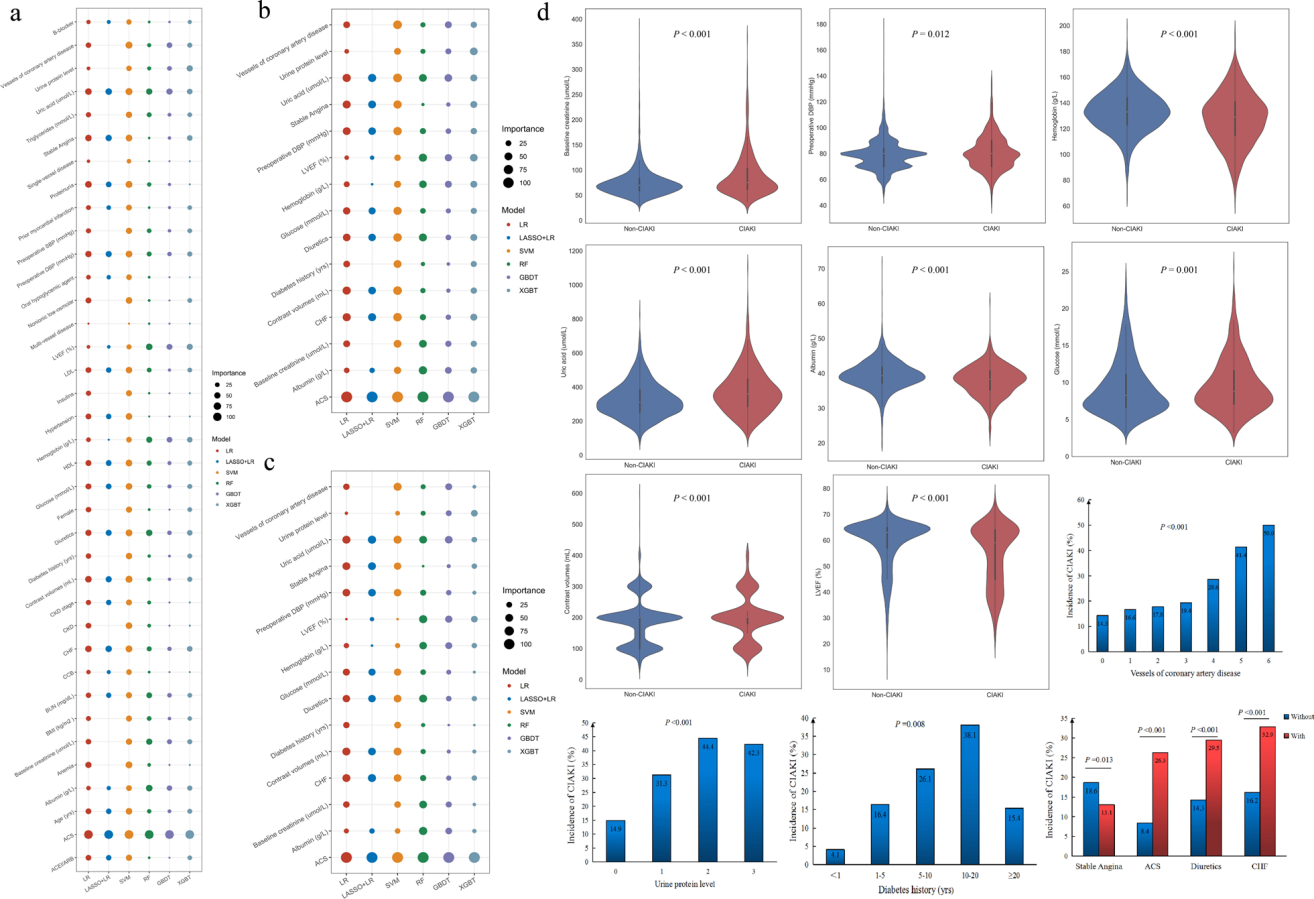
Importance of features in ML models and subgroup analysis of BCPMD features. **a** Importance rank of all features for identifying CIAKI in diabetes included in the 6 different ML models. The size of the circles represents the degree of contribution to CIAKI. The different colors of circles represent different models. **b** A subset of (**a**), showed the degree of contributions of 15 features in BCPMD for identifying CIAKI relative to all features of (**a**) in 6 different models. **c** Degree of contributions of 15 features in BCPMD for identifying CIAKI relative to each other in 6 different models. **d** Subgroup analysis of BCPMD features. Violin plots show the distribution of continuous features included in BCPMD between CIAKI patients (n = 634) and non-CIAKI patients (n = 2880). The thick black line in the middle represents the interquartile range [IQR]. The thin black line represents the 95% confidence interval. The white point is the median, and the shapes on both sides represent the distribution density of the data. The median [IQR] of the features shown in Fig. **d** are listed in Additional file 1: Table S5. Bar plots show the incidence of CIAKI among the categorical features of BCPMD. BCPMD, brief CIAKI prediction model for diabetes based on the XGBT model

During the study, 61 essential characteristics from electronic medical records were chosen, including 5 demographic data, 13 medical history characteristics, 9 intraoperative indicators, 6 preoperative medications, 16 preoperative laboratory tests, and 6 postoperative serum creatinine and postoperative blood urea nitrogen at 24, 48, and 72 h. In addition, 6 clinical endpoint variables were included. After data cleaning, missing values greater than or equal to 11% (Killip classification, glycated hemoglobin and NYHA classification) were removed. At the same time, collinear features were removed, including height, weight, nonionic iso-osmolar and total cholesterol. Since this study used pre-CAG + PCI and intraoperative indicators to predict the occurrence of postoperative CIAKI, we removed postoperative serum creatinine and postoperative blood urea nitrogen at 24, 48, and 72 h. We retained preoperative serum creatinine and preoperative blood urea nitrogen. In addition, 6 clinical endpoint variables occurred postoperatively, and we did not include them as risk factors As a result, 37 features, including 20 categorical features and 17 continuous variables, were retained in the training cohort to establish ML models. Further, we screened 23 features by the LASSO + LR model for CIAKI (Additional file 1**: **Figure S2). We also showed the top 20 features for CIAKI in each ML model (Additional file 1: Figure S3). Figure 2a illustrated the scaled importance rank of all features in the six ML models for identifying CIAKI risk in diabetic patients. Figure 2b, a subset of Fig. 2a, showed the importance of the final 15 variables screened in the six ML models. Figure 2c showed the relative importance of the 15 variables in Fig. 2b in the six ML models. As shown in Figs. 2b and c, ACS presented the most crucial feature in all ML models.

### Model performance

**Fig. 3:**
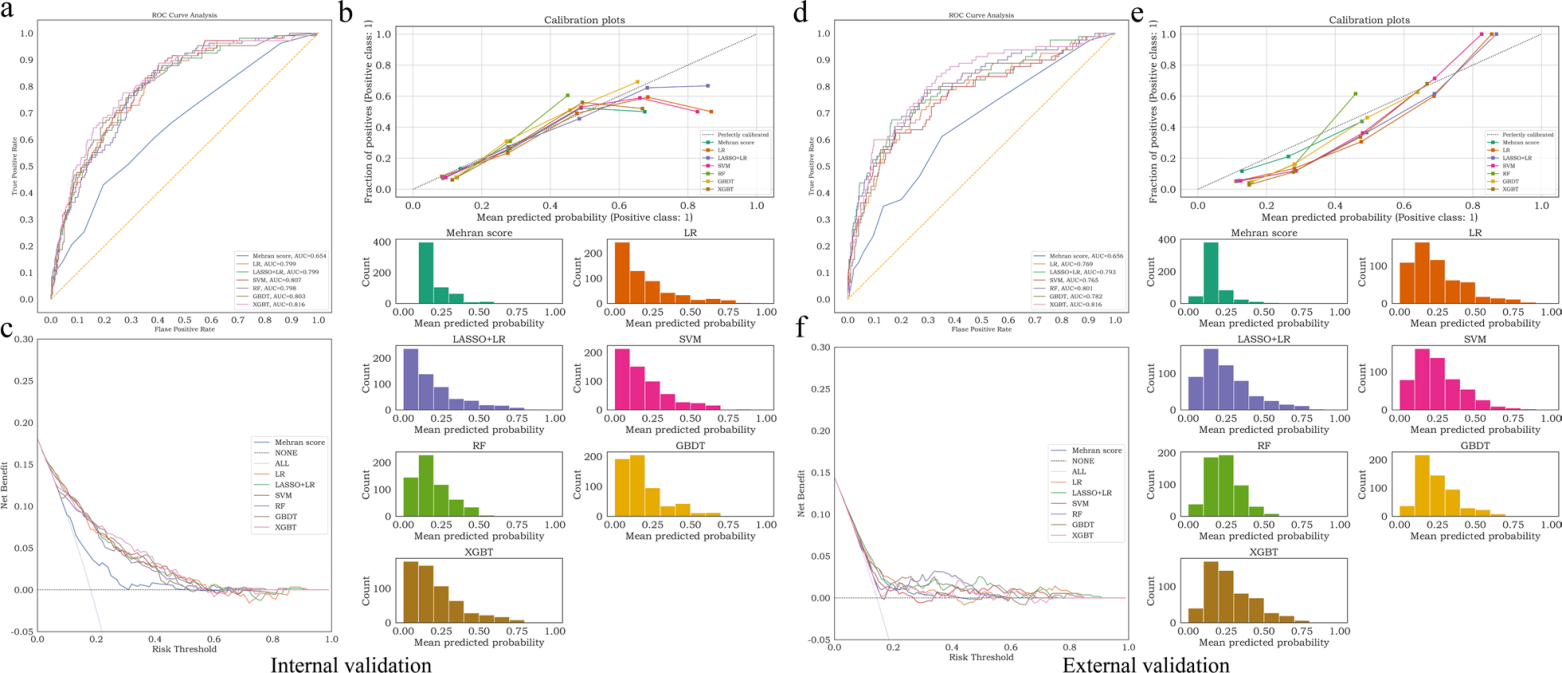
Predictive performance of ML models in the internal validation cohort and external cohorts. **a** Comparison of AUCs among the Mehran score, LR, LASSO + LR, SVM, RF, GBDT and XGBT models in the internal validation cohort. **b** Calibration curve of Mehran score (ECE = 0.046), LR (ECE = 0.037), LASSO + LR (ECE = 0.046), SVM (ECE = 0.055), RF (ECE = 0.068), GBDT (ECE = 0.061) and XGBT (ECE = 0.054) models in the internal validation cohort. A smaller value of the expected calibration error (ECE) represents better calibration. **c** Decision curve analysis (DCA) of the Mehran score, LR, LASSO + LR, SVM, RF, GBDT and XGBT models in the internal validation cohort. The risk threshold represents the cutoff above which a patient may develop CIAKI. Net benefit is a weighted measure between true and false positives depending on the threshold. The None line and ALL line intersect at the point of risk threshold = 0.181, which also represents the internal CIAKI incidence. DCA of models above the NONE and ALL lines is considered clinically useful. **d** Comparison of AUCs among the Mehran score, LR, LASSO + LR, SVM, RF, GBDT and XGBT models in the external validation cohort. **e** Calibration curve of Mehran score (ECE = 0.048), LR (ECE = 0.111), LASSO + LR (ECE = 0.114), SVM (ECE = 0.116), RF (ECE = 0.125), GBDT (ECE = 0.116) and XGBT (ECE = 0.143) models in the external validation cohort. **f** DCA of the Mehran score, LR, LASSO + LR, SVM, RF, GBDT and XGBT models in the external validation cohort

In the internal validation cohort, all ML models achieved higher AUC than Mehran score, of which the XGBT model achieved the best AUC (0.816, 95% CI 0.777 to 0.853) (Fig. 3a). Additionally, all of the ML models outperformed the Mehran score in terms of *F*_*1*_ score, accurary, sensitivity, spensitivity, PPV and NPV. (Table 3). The XGBT model demonstrated superior performance than the other 4 ML models. The calibration curves of the 5 ML models and Mehran score were shown in Fig. 3b**.** The DCA indicated that the risk threshold of the XGBT model ranged from 0.20 to 0.50, which was superior to the ranges associated with other models (Fig. 3c). Additionally, the net benefit of the XGBT, RF, LASSO + LR and LR were optimistic when the risk threshold was in the range of 0 to 0.55.

**Table 3:** Prediction performance of CIAKI models in the internal and external validation cohorts

Classifier	AUC (%) (95% CI)	Accuracy (%) (95% CI)	Sensitivity (%) (95% CI)	Specificity (%) (95% CI)	PPV (%) (95% CI)	NPV (%) (95% CI)	*F*_*1*_ score (%) (95% CI)
Internal validation performance							
Mehran score	65.41 (60.85–69.93)	67.57 (64.53–70.78)	50.47 (42.57–58.68)	71.34 (67.92–74.59)	27.98 (22.63–33.17)	86.72 (83.95–89.43)	36.00 (30.07–41.82)
LR	79.90 (75.95–83.32)	78.21 (75.34–81.08)	57.01 (49.04–64.55)	82.89 (80.00–85.66)	42.36 (35.62–48.82)	89.73 (87.39–92.01)	48.61 (41.92–54.62)
LASSO + LR	79.92 (75.91–83.44)	78.21 (75.68–80.74)	56.07 (47.97–63.46)	83.09 (80.21–85.71)	42.25 (35.17–48.63)	89.56 (87.17–91.96)	48.19 (41.30–54.10)
SVM	80.73 (76.78–83.99)	79.05 (76.35–81.76)	55.14 (47.32–62.83)	84.33 (81.57–86.95)	43.70 (36.57–50.72)	89.50 (87.26–91.81)	48.76 (41.88–55.23)
RF	79.76 (76.07–83.06)	78.89 (76.18–81.59)	53.27 (45.61–61.11)	84.54 (81.78–86.95)	43.18 (36.17–50.00)	89.13 (86.75–91.54)	47.70 (40.98–53.85)
GBDT	80.28 (76.49–83.98)	80.41 (77.70–83.11)	57.01 (49.51–64.81)	85.57 (82.85–88.02)	46.56 (39.31–53.78)	90.02 (87.78–92.36)	51.26 (44.64–57.52)
XGBT	81.59 (77.71–85.33)	81.08 (78.55–83.78)	54.21 (46.23–62.26)	87.01 (84.66–89.41)	47.93 (40.59–55.36)	89.60 (87.32–91.97)	50.88 (44.05–57.26)
External validation performance							
Mehran score	65.59 (60.18–71.10)	73.83 (70.76–76.71)	37.50 (28.57–46.84)	79.96 (76.89–83.05)	24.00 (17.74–30.66)	88.34 (85.75–90.76)	29.27 (22.55–36.36)
LR	76.94 (71.84–82.03)	72.02 (68.95–75.09)	71.25 (62.82–79.22)	72.15 (68.92–75.48)	30.16 (25.00–35.67)	93.70 (91.42–95.71)	42.38 (36.36–48.63)
LASSO + LR	79.31 (74.40–84.17)	72.20 (69.13–75.27)	73.75 (65.17–82.05)	71.94 (68.78–75.26)	30.73 (25.77–36.04)	94.20 (91.98–96.21)	43.38 (37.36–49.64)
SVM	76.47 (71.14–81.58)	70.58 (67.51–73.65)	65.00 (56.32–73.56)	71.52 (68.35–74.79)	27.81 (22.70–33.16)	92.37 (90.00–94.47)	38.95 (32.96–44.85)
RF	80.10 (75.33–84.64)	72.56 (69.49–75.63)	75.00 (66.67–82.89)	72.15 (68.90–75.48)	31.25 (26.02–36.95)	94.48 (92.39–96.46)	44.12 (38.02–50.52)
GBDT	78.16 (73.27–83.01)	72.38 (69.31–75.27)	76.25 (68.24–83.75)	71.73 (68.39–74.95)	31.28 (26.09–36.59)	94.71 (92.76–96.47)	44.36 (38.38–50.49)
XGBT	81.65 (77.04–86.09)	75.99 (73.10–79.06)	72.50 (64.00–80.82)	76.58 (73.24–79.74)	34.32 (28.57–40.34)	94.29 (92.19–96.16)	46.59 (40.17–52.80)

*LR* logistic regression, *LASSO* least absolute shrinkage and selection operator, *SVM* support vector machine, *RF* random forest, *GBDT* gradient boosted decision trees, *XGBT* extreme gradient boosting trees, *AUC* area under the curve, *PPV* positive predictive value, *NPV* negative predictive value, *95% CI* 95% confidence interval

The AUC performance in the cohort of external validation was comparable to that in the cohort of internal validation. The better performance of the ML models than the Mehran score remained consistent. XGBT model achieved better AUC (0.816, 95% CI 0.770 to 0.861) than others (Fig. 3d). Furthermore, the calibration curves of all models still performed well (Fig. 3e**)**. According to the DCA, the XGBT model provided a clinical net benefit when models ranged from 0.10 to 0.65 (Fig. 3f**)**.

### The best prediction model determination

**Fig. 4:**
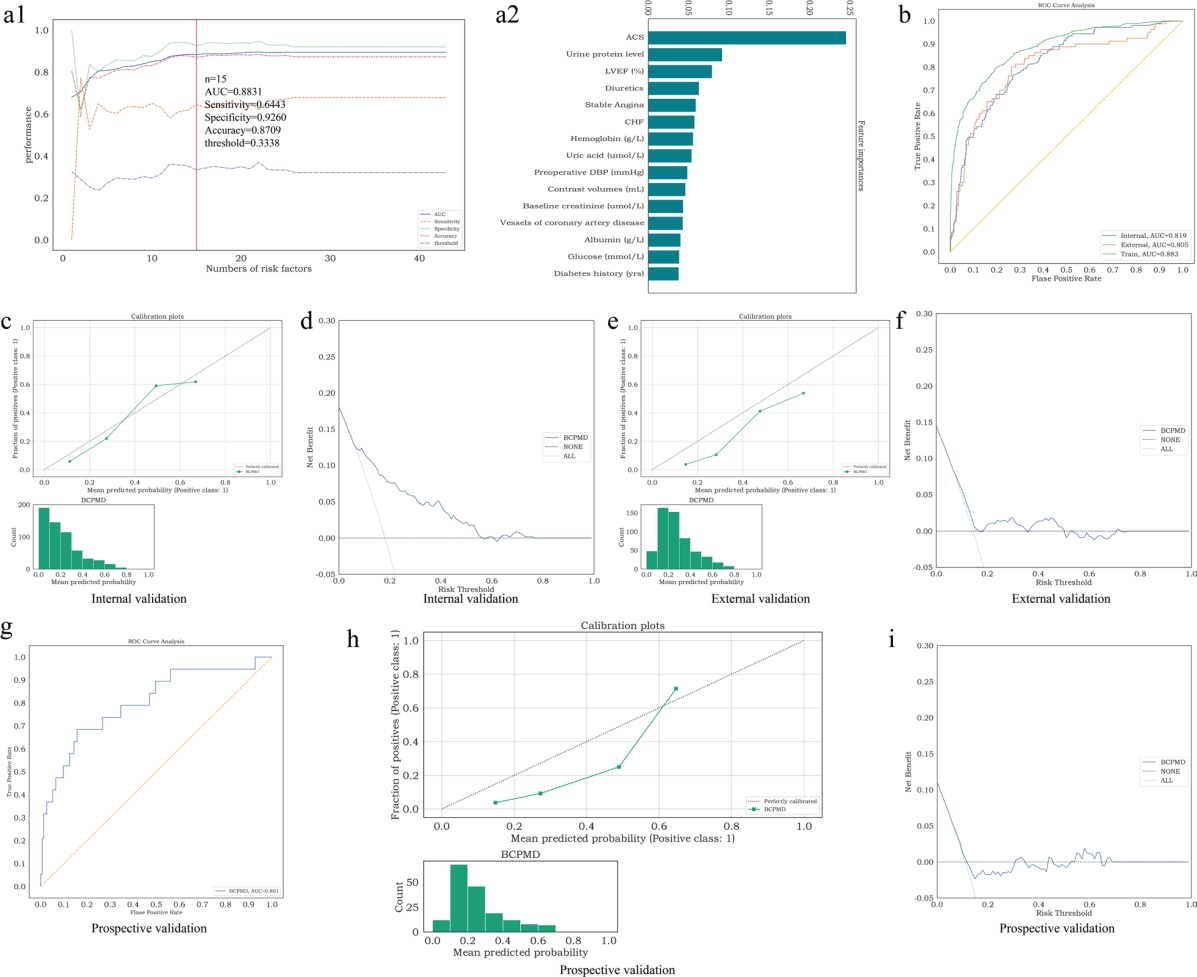
Feature selection of BCPMD and predictive performance of BCPMD in the internal, external and prospective validation cohorts. (**a1**) The performance of the XGBT model trained with n features. The features were sorted according to the feature importance in descent order in the training set. The performances of AUC, sensitivity, specificity and accuracy corresponding to the top n number of features were shown in the figure, and we finally determine the features and the corresponding threshold for judging CIAKI according to the performance of AUC and clinical convenience and practice. When n = 15, the AUC was 0.8831, sensitivity was 0.6443, specificity was 0.9260, accuracy was 0.8709 and the threshold was 0.3338. (**a2**) The importance ranking of the first 15 features of the XGBT model, which is called the brief CIAKI prediction model for diabetes (BCPMD). **b** AUCs of BCPMD in the internal validation, external validation and training cohorts. **c** Calibration curve of BCPMD (ECE = 0.073) in the internal validation cohort. **d** DCA of BCPMD in the internal validation cohort. The net benefit was positive when the risk threshold ranged from 0.10 to 0.78. **e** Calibration curve of BCPMD (ECE = 0.135) in the external validation cohort. **f** DCA of BCPMD in the external validation cohort. The net benefit was positive when the risk threshold ranged from 0.20 to 0.50. **g** AUC of BCPMD in the prospective validation cohort. **h** Calibration curve of BCPMD (ECE = 0.155) in the prospective validation cohort. **i** DCA of BCPMD in the prospective validation cohort. The net benefit was positive when the risk threshold ranged from 0.30 to 0.35, 0.42 to 0.44 and 0.55 to 0.70

Among all the models, XGBT achieved the best AUC in both internal and external validation cohorts. In general, the AUC of the model will increase as more features are selected. Nevertheless, adding more features does not improve clinical practice. To discover the significant features, we sorted the importance of XGBT features in descent order in the training set. The performances of AUC, sensitivity, specificity and accuracy corresponding to the top n number of features in XGBT were shown in Fig. 4a1. When the number of features was increased to the top 15, the AUC rose to 0.883, and the accuracy and sensitivity had significant improvements. With the number of features growing to 22, the AUC steadily reached its maximum which was 0.896. However, the sensitivity had a small decrease from 0.644 to 0.611 when the number was from the top 15 to 22. The performance of AUC, specificity, sensitivity and accuracy tended to be stable and no longer changed after more than 27 features. Considering more features were not beneficial to clinical use and practice, and the AUCs after 15 variables were overall stable, to facilitate the application in clinical practice, we selected the top 15 critical variables as the brief CIAKI prediction model for diabetes, called the BCPMD model: ACS, Urine protein level, Diuretics, left ventricular ejection fraction (LVEF)(%), Hemoglobin, CHF, Stable Angina, Uric acid, Preoperative DBP, Contrast Volumes, Albumin, Baseline creatinine, Vessels of coronary artery disease, Glucose and Diabetes history (Fig. 4a1 and a2). The corresponding risk threshold of BCPMD was 0.3338 which was based on maximizing the *F*_*1*_ score. Violin plots were analyzed to demonstrate the distribution of 8 continuous characteristics contained in BCPMD between CIAKI patients (n = 634) and non-CIAKI patients (n = 2880) (Fig. 2d and Additional file 1: Table S5). Also, relationships between 7 categorical features and CIAKI were observed in Fig. 2d. Besides, The AUCs of the BCPMD for CIAKI in the cohort of training, internal validation, and external validation were 0.883 (95% CI 0.867–0.898), 0.819 (95% CI 0.783–0.855), and 0.805 (95% CI 0.755–0.850), respectively (Fig. 4b). The expected calibration errors (ECE) in calibration curves of BCPMD were 0.073 for the cohort of internal validation and 0.135 for external validation (Fig. 4c and e). The net benefits of the BCPMD in the cohort of external validation were reduced than in the cohort of internal validation (Fig. 4d and f**)**.

Table 4 displayed the BCPMD’s prospective predictive performance. AUC, accuracy, sensitivity, specificity, PPV, NPV, and *F*_*1*_ scores of BCPMD were 0.801 (95% CI 0.688–0.887), 0.826 (95% CI 0.779–0.866), 0.684 (95% CI 0.500–0.846), 0.843 (95% CI 0.793–0.887), 0.351 (95% CI 0.219–0.485), 0.956 (95% CI 0.924–0.979), 0.464 (95% CI 0.311–0.586), respectively. Moreover, CIAKI occurred in 19/172 (11.0%) in the prospective cohort. Of the 19 patients with true CIAKI, BCPMD correctly predicted 13 patients. Additional file 1**: **Table S6 displayed the prospective validation cohort’s basic characteristics.

**Table 4:** Prediction performance of BCPMD in the internal, external and prospective validation cohorts

BCPMD	AUC (%) (95% CI)	Accuracy (%) (95% CI)	Sensitivity (%) (95% CI)	Specificity (%) (95% CI)	PPV (%) (95% CI)	NPV (%) (95% CI)	*F*_*1*_ score (%) (95% CI)
Internal validation performance	81.93 (78.25–85.46)	81.76 (79.22–84.46)	54.21 (46.49–62.39)	87.84 (85.41–90.04)	49.57 (41.98–57.14)	89.68 (87.37–91.98)	51.79 (45.33–58.33)
External validation performance	80.48 (75.49–85.03)	76.90 (74.01–79.78)	70.00 (60.98–78.38)	78.06 (75.00–81.01)	35.00 (29.05–41.21)	93.91 (91.69–95.84)	46.67 (40.00–53.15)
Prospective validation performance	80.08 (68.83–88.70)	82.56 (77.91–86.63)	68.42 (50.00–84.62)	84.31 (79.31–88.74)	35.14 (21.88–48.48)	95.56 (92.41–97.92)	46.43 (31.11–58.62)

*BCPMD* brief CIAKI prediction model for diabetes, *AUC* area under the curve, *PPV* positive predictive value, *NPV* negative predictive value, *95% CI* 95% confidence interval

### SHAP values evaluate feature importance

**Fig. 5:**
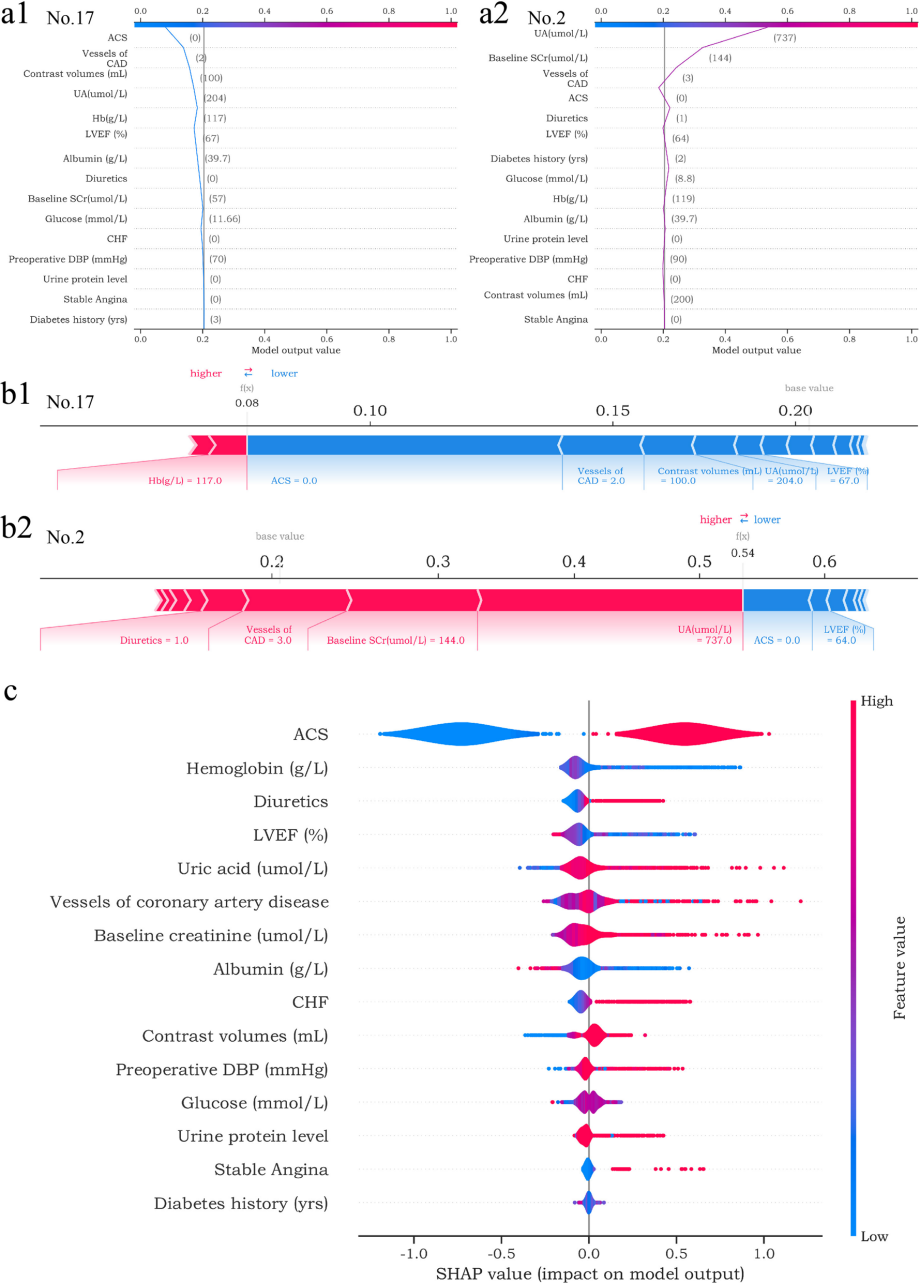
SHAP explains the contributions of BCPMD features to CIAKI. (**a1**-**a2**) SHAP decision plot of the 2 patients (No. 17 and No. 2). The plots depict the decision path for predicting CIAKI and can better visualize the impact of each feature on the occurrence of CIAKI at the individual level. a1 shows the example of patient No. 17 predicted to be non-CIAKI. a2 shows the example of patient No. 2 predicted to have CIAKI. (**b1**-**b2**) SHAP force plot of the 2 patients (No. 17 and No. 2). The features shown in red represent a higher risk of CIAKI, while the features shown in blue represent a lower risk. The plots help physicians identify the main features in the model that have high decision power at the individual level. **c** SHAP summary plot. Sort features according to the sum of all samples SHAP values in the training cohort. The SHAP summary plot demonstrates the distribution of each feature influence on the model output. The color bar on the right indicates the relative size of each feature. Red dots indicate high values, and blue dots indicate low values. The violin plots arranged on the median line represent the aggregation of each case in the training cohort. The distance between the upper and lower margins of the violin plots represents the number of cases with the same SHAP values offered by this feature

We explained the BCPMD through the SHAP diagram. After inputting each variable, the model’s positive or negative contribution could be observed (Fig. 5c). The SHAP summary plot demonstrated that ACS, hemoglobin, diuretics, LVEF (%) and uric acid (umol/L) ranked as the top 5 important features. Moreover, the SHAP plot revealed that ACS, lower hemoglobin (g/L), using diuretics, lower LVEF (%) and higher uric acid (umol/L) were correlated with a greater SHAP value generated in BCPMD, implying a higher risk of CIAKI. Figure 5a showed two cases (Patient No.2, No.17) by SHAP decision plot, which simulated the path of each feature decision. In addition, the different feature values of BCPMD represented different positive and negative contributions to the final SHAP value output. Red values represented higher risk factors, and blue values represented lower risk factors (Fig. 5b). It reflects the personalized interpretation function of SHAP and helps physicians make clinical decisions on the individual level.

### CIAKI Web calculator development

**Fig. 6:**
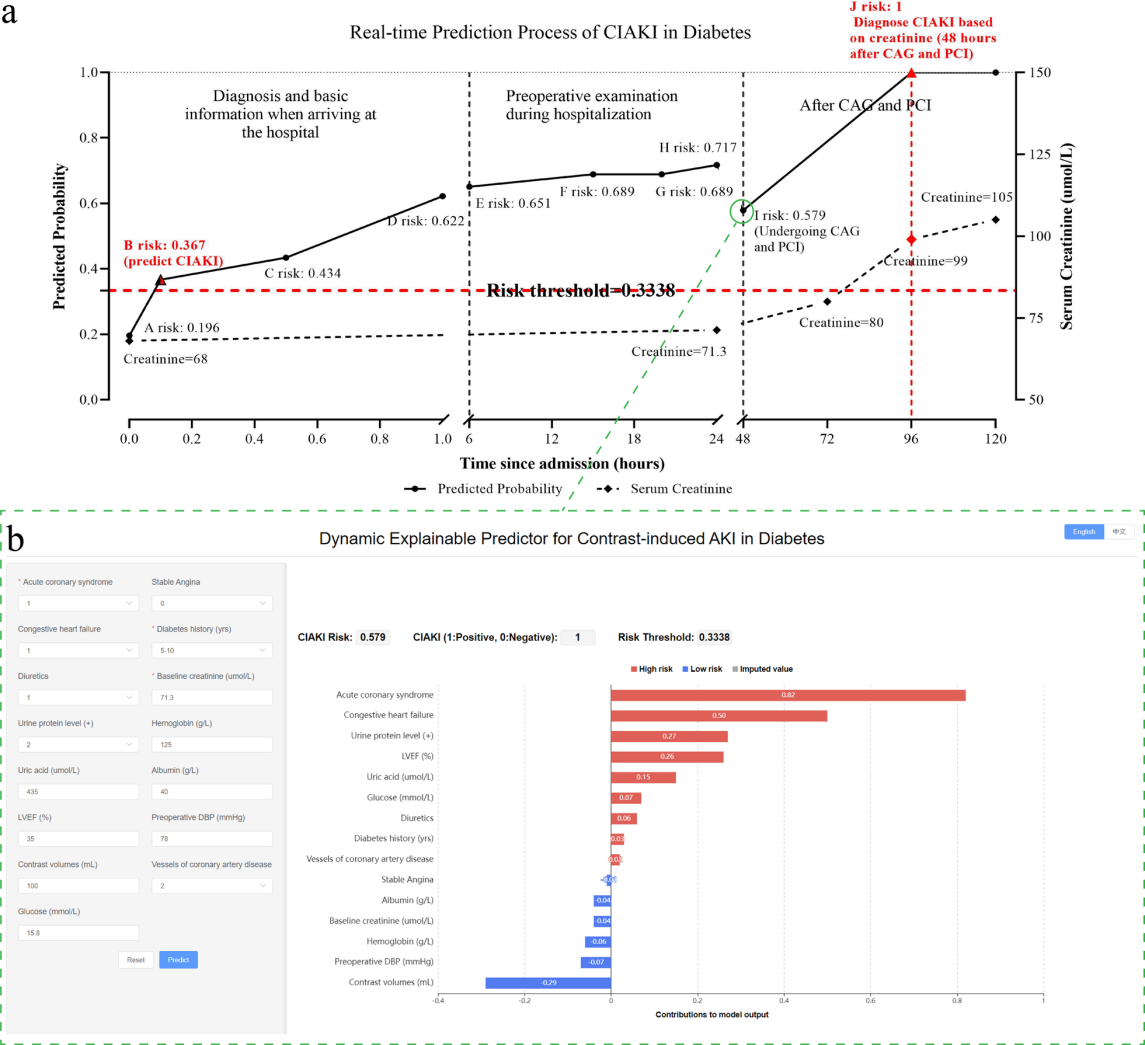
Real-time prediction process of CIAKI in diabetes based on the web calculator platform. **a** An example of CIAKI prediction in one hospitalized patient. When a patient arrived at the hospital, the doctor obtained the basic information of the patient and made a diagnosis within 0–6 h. At point A, we knew that ACS was 1, stable angina was 0, his previous serum creatinine was 68 µmol/L, and his diabetes history was 5–10 years. According to the CIAKI web predictive platform, other missing values were filled, and we calculated that the risk was 0.196. At point B, we knew that the CHF was 1, and the risk rose to 0.367 and predicted CIAKI occurrence (risk threshold was 0.3338). At point C, the patient’s preoperative DBP was 78 mmHg, glucose was 15.8 mmol/L and the risk rose to 0.434. At point D, the patient took diuretics, and the risk was 0.622. During the period of 6 h-24 h, the patient underwent preoperative examination. At E, hemoglobin was 125 g/L, and the risk was 0.651. At point F, the urine protein level was 2 + , and the risk was 0.689. At point G, LVEF was 35%, and the risk was still 0.689. At point H, uric acid was 435 µmol/L, albumin was 40 g/L, serum creatinine was 71.3 µmol/L, and the risk was 0.717. When the patient arrived 48 h after admission (point I), he underwent CAG and PCI and used contrast volumes of 100 mL, and he had 2 vessels of coronary artery disease; the risk was 0.579. Creatinine was examined at 24 h, 48 h and 72 h after CAG and PCI, and the real occurrence of CIAKI was diagnosed at 48 h after CAG and PCI (point J). **b** All features of BCPMD were known at point I, and the model output the risk value using the dynamic explainable CIAKI predictor. In this example, our web platform identified patients with possible CIAKI within 1 h of admission

Based on the BCPMD, we built a dynamic and explainable website to calculate the risk of CIAKI in diabetes. The URL is here: http://49.51.70.39/**.** When a patient plans to undergo CAG and PCI, the physician can enter the associated risk factor values into the website, which will immediately produce the projected risk values for CIAKI. Furthermore, the risk of CIAKI was judged as negative or positive according to the threshold of 0.3338 on the platform. Moreover, we used the missForest method to impute the missing data to predict the risk of CIAKI even when features are unknown. Notably, we developed a dynamic and explainable waterfall diagram to show the positive or negative contribution of different risk factor values, in which red presents higher risk and blue presents lower risk. Figure 6 showed an example that our web calculator predicted CIAKI in a case within 1 h of admission.

## Discussion

In this study, we employed ML algorithms to develop an innovative prediction tool. Compared to Mehran risk scores, our results showed that ML models were superior to traditional logistic regression. Notably, in both the cohort of internal and external validation, the XGBT model performed best. Further, we determined the top 15 important predictors in the XGBT model as BCPMD model variables as these variables can be collected easily in medical activities. Similarly, AUC for CIAKI in the cohorts of internal validation, external validation, and prospective validation was shown by BCPMD to be 0.819 (95% CI 0.783–0.855), 0.805 (95% CI 0.755–0.850) and 0.801 (95% CI 0.688–0.887), respectively. In addition, we constructed SHAP to provide personalized interpretation for each patient. An online web risk calculator model of CIAKI in diabetes was then established to predict the occurrence of CIAKI within 1 h when patients arrived at the hospital.

The previous study indicated Mehran’s score could predict CIAKI with an AUC of 0.67 in the validation cohort [9]. Our results verified the AUC of Mehran score was 0.654 in the cohort of internal validation and 0.656 in external validation for CIAKI in patients with diabetes. Mehran score models were updated in 2021, with model 1 including indicators before CAG, and model 2 adding procedural features, giving a better AUC of 0.84 [22]. However, with the development of biomarkers and algorithms, ML technology is gradually emerging as a better tool for establishing prediction models. Yin et al. [23] constructed a CIAKI prediction model using 13 preprocedural indicators through an RF algorithm, revealing an AUC of 0.907 and an accuracy of 80.8%. Other researchers also found that GBDT [24] and RNN [25] could perform well in predicting CIAKI. Moreover, Sun et al. [26] exhibited that in patients with ACS, the LASSO + LR-based nomogram model provided a better prediction of CIAKI than the Mehran score (AUC was 0.835 and 0.762, respectively). According to our results, in diabetic patients, ML models (including LASSO + LR, GBDT, XGBT, and SVM) demonstrated better discriminative power than traditional LR and Mehran score in developing predictive models. Additionally, our data displayed that XGBT performed best, which was an ensemble of weak prediction trees [27]. The XGBT algorithms can capture complex relationships in data without explicit specification of higher-order interactions and nonlinear functions [28]. Furthermore, XGBT algorithms prevent overfitting through cross-validation and regularization [29].

The BCPMD model included 15 features, which were easily accessible in clinical activities. Although the 15 features were readily accessible, missing data could still occur in different regions or circumstances, affecting the model’s performance and delaying the prediction time. As a result, we adopted missForest [30] to handle mixed-type data with both missing continuous and categorical patient variables to make our web predictive tool perform well.

Notably, our model suggested that ACS was the most significant risk factor for CIAKI in diabetic individuals, consistent with current studies [31–33]. In addition to the signal pathway regulation and contrast medium’s harmful effects on renal tubular cells [34], ACS may have a comparable mode of action with diabetes, leading to the superposition of kidney injury. On the one hand, they both affect renal perfusion. Patients with ACS often have unstable hemodynamics. In the case of cardiac vascular stenosis, cardiac ejection function is impaired, and hypotension occurs, which may result in decreased renal perfusion and kidney injury [35]. Likewise, acute myocardial ischemia can activate renin angiotensin aldosterone system (RAAS). Vasopressin, catecholamine and interleukin are produced, and the level of nitric oxide is reduced, damaging endothelial cells and bringing about decreased renal blood flow [36, 37]. On the other hand, ACS can give rise to kidney inflammation and oxidative stress damage, like diabetes [38, 39].

Additionally, our results revealed that hyperuricemia constituted a significant risk factor for CIAKI in diabetes. A recent study from China proved that hyperuricemia was associated with CIAKI (OR = 2.363, 95% CI 1.653–3.377, *P* < 0.001) [40]. What’s more, it was also shown that patients with uric acid levels above 8.0 mg/dL not only had a greater risk of CIAKI but also an increased risk of hemodialysis [41]. Uric acid can promote oxidative stress and release a variety of proinflammatory factors, resulting in renal vasoconstriction and endothelial dysfunction [42]. At the same time, contrast agents can give rise to acute uricosuria [43], further aggravating kidney injury. Besides, diuretics were one of the important factors in the model. This may be because diuretics can accelerate the excretion of iodine and improve urine viscosity [44]. Whereas more and more studies believed that diuretics are independent predictors of CIAKI in recent years [45, 46]. The National Kidney Foundation and the American College of Radiology proposed that it was not recommended to use drugs that can affect renal function within 48 h before and after iodine contrast agents, including diuretics [47]. Considering the hypoxia and inflammatory reaction induced by diuretics, using diuretics during the perioperative period of PCI may be a potential risk of CIAKI [48]. Our study also confirmed the increased risk of CIAKI among patients suffering from heart failure, worse renal function, anemia, poor blood glucose control and more contrast volumes, underlining the need for early prevention strategies for these patients at high risk.

Of note, our web CIAKI risk calculator could be used as a guide for clinicians compared with previous studies that only stayed in constructing models, lacking practical value. Evidence has shown that early clinical intervention could improve CIAKI patients’ outcomes [49, 50]. The time window between evidence of increased CIAKI risk in the prediction platform and the occurrence of clinical CIAKI is an ideal period for clinical intervention. When combining the platform’s prediction and early intervention, the risk of CIAKI is expected to be reduced.

Our study has several strengths, the first of which was generalizability. We assessed the BCPMD model in multi-centre hospitals and prospectively constructed the web platform based on BCPMD. Our results also showed that despite the difference in our data distribution in the external set, it did not affect the model’s predictive ability, indicating that the BCPMD model is generalizable. Secondly, the feature of BCPMD was readily accessible in routine clinical practice. We also found that it was not the greater the number of predictor variables, the higher the model’s prediction ability. Therefore, we screened out a certain number of optimal subsets according to the model effect of different feature numbers to make the model more efficient and straightforward. Thirdly, our model can be used for clinical practice. We developed a dynamically interpretable prediction web platform for the first time. Meanwhile, we set the missing value filling for the platform. Additionally, considering the ML models’ black-box flaws, we used SHAP to explain whether features contributed positively or negatively to ML models, which can explain how each characteristic affects the overall forecast of the model and how our model features affect CIAKI at the individual level. Our web calculator provides a tool that can real-time predict high-risk CIAKI patients and helps clinicians simply and intuitively understand how different values of a single feature affect the model's predictions, which can be as a reference for other disease models.

We also have some limitations in our research. Firstly, 30% of our CAG + PCI patients were excluded from the inclusion criteria. Although most of our characteristics did not differ between excluded patients and included patients, there are still some characteristics that we did not pay attention to that might have possible bias. Therefore, a large sample size of data is needed for verification in the future. Secondly, even though our model’s AUC in the prospective validation set was performing well, we observed that not all risk thresholds were beneficial for patients. In the prospective validation set, a risk threshold lower than 0.30 has no benefit. However, it can identify more lower-risk CIAKI patients, who can give routine interventions such as closely detecting the serum creatinine. In the risk threshold of 0.55 to 0.70, patients with CIAKI can benefit and be identified more accurately. More comprehensive intervention methods, such as adequate hydration, should be given to these high-risk patients. However, using a higher risk threshold means part of CIAKI patients may not be identified ahead of time. It needs to be set according to the patient characteristics of different institutions. Thirdly, we still used serum creatinine for the definition of CIAKI. More early diagnostic markers and clinical features could be added to increase the prediction probability of CIAKI in the future.

## Conclusions

In conclusion, we developed a web tool based on the BCPMD model that could identify high-risk CIAKI patients in diabetes and accurately stratify the risk of CIAKI. In the future, early kidney injury prevention combined with artificial intelligence are expected to improve outcomes in patients with CIAKI.

## Supplementary Information

**Additional file 1: Methods**. Description of the six ML models. **Table S1**. Baseline characteristics between included and excluded patients. **Table S2**. Baseline characteristics of patients in the three groups. **Table S3**. Ten-fold cross-validation results of AUC and accuracy in models. **Table S4**. Model performance using different balancing methods. **Table S5**. The median [IQR] of the continuous features in BCPMD. **Table S6**. Baseline characteristics of patients in the prospective cohort. **Figure S1**. Ten-fold cross-validation results of AUC and accuracy in models. **Figure S2**. Feature screening process of LASSO. (a) Lasso ten-fold cross-validation determines the number of important features according to the binomial deviation (λ=0.005774419, n=23). (b) The dynamic change in risk factors with the penalty coefficient; the vertical line indicates the optimal λ (n=23). **Figure S3**. The top 20 features of ML models. **Figure S4**. The prediction process of a patient on the dynamic explainable CIAKI risk calculator.

## Data Availability

The data that support the findings of this study are available from the corresponding author upon reasonable request.

## Abbreviations

PCI: Percutaneous coronary intervention
CIAKI: Contrast-induced acute kidney injury
ML: Machine learning
RF: Random forest
GBDT: Gradient-boosted decision trees
LR: Logistic regression
LASSO: Least absolute shrinkage and selection operator
XGBT: Extreme gradient boosting trees
SVM: Support vector machine
AUC: Area under the curve
PPV: Positive predictive value
NPV: Negative predictive value
DCA: Decision curve analysis
95% CI: 95% Confidence interval
BCPMD: Brief CIAKI prediction model for diabetes
SHAP: Shapley additive explanations
ACS: Acute coronary syndromes
LVEF: Left ventricular ejection fraction
BMI: Body mass index
CKD: Chronic kidney disease
CHF: Congestive heart failure
SBP: Systolic blood pressure
DBP: Diastolic blood pressure
CCB: Calcium channel blocker
ACEI: Angiotensin-converting enzyme inhibitor
ARB: Angiotensin receptor blocker
eGFR: Estimated glomerular filtration rate
HDL: High-density lipoprotein
LDL: Low-density lipoprotein
DM: Diabetes mellitus
CAG: Coronary angiography
TRIPOD: Transparent reporting of a multivariable prediction model for individual prognosis or diagnosis
STROBE: Strengthening the reporting of observational studies in epidemiology
CMSC: Contrast media safety committee
NYHA: New York Heart Association
VIF: Variance inflation factor
SMOTE: Synthetic minority oversampling technique
ADASYN: Adaptive synthetic technique
IABP: Intra-arterial balloon pump
IQR: Interquartile range
