# Supplementary material for: A novel explainable online calculator for contrast-induced AKI in diabetics: a multi-centre validation and prospective evaluation study
Source: J Transl Med. 2023 Jul 31;21:517. doi: 10.1186/s12967-023-04387-x (PMC10391987; doi:10.1186/s12967-023-04387-x)
Supplement: Supplementary file 1 — Additional file 1: Methods. Description of the six ML models. Table S1. Baseline characteristics between included and excluded patients. Table S2. Baseline characteristics of patients in the three groups. Table S3. Ten-fold cross-validation results of AUC and accuracy in models. Table S4. Model performance using different balancing methods. Table S5. The median [IQR] of the continuous features in BCPMD. Table S6. Baseline characteristics of patients in the prospective cohort. Figure S1. Ten-fold cross-validation results of AUC and accuracy in models. Figure S2. Feature screening process of LASSO. (a) Lasso ten-fold cross-validation determines the number of important features according to the binomial deviation (λ=0.005774419, n=23). (b) The dynamic change in risk factors with the penalty coefficient; the vertical line indicates the optimal λ (n=23). Figure S3. The top 20 features of ML models. Figure S4. The prediction process of a patient on the dynamic explainable CIAKI risk calculator. [file 12967_2023_4387_MOESM1_ESM.doc]

**Supplementary Methods. Description of the six machine learning models**

**Logistic Regression (LR)**

LR is used to predict the probability of events with discrete dependent variables. The model uses the sigmoid function to predict a logistic transformation of the probabilities for each class in the dependent variable. We trained an LR model on the training set of all factors. Moreover, an L2 penalty term was added to the model to avoid overfitting.

**Least Absolute Shrinkage and Selection Operator with LR (LASSO+LR)**

LASSO is a regression analysis method that performs both variable selection and regularization to enhance the accuracy and interpretability of the predictive model. It obtains a more refined model by constructing a penalty function. The lambda parameter used for the model was a value of 0.005774419, whose binomial deviation was the lowest one in the training set according to ten-fold cross-validation and reduced the count of factors to 23. An LR model was then trained on the training set of these 23 factors with no penalty added.

**Support Vector Machine (SVM)**

SVM is a binary classification model whose basic model is a linear classifier with the largest margin defined on the feature space, and the largest margin distinguishes it from a perceptron; SVM also includes kernel tricks, which make it essentially a nonlinear classifier. We developed the SVM models using a linear basis function kernel with the “class_weight” parameter set to “balanced” and regularization parameter C set to 0.1, which achieved the highest AUC in the training set according to ten-fold cross-validation. We trained an SVM model on the training set of all factors.

**Random Forest (RF)**

RF is a classification algorithm that applies an ensemble of decision trees and a bootstrapping method to sample training data and split nodes in each tree. The algorithm was deployed in our data set with 60 trees in the forest using the Gini criterion for splitting, and the maximum depth of the tree was 5. These hyperparameters were also optimized through grid search and ten-fold cross-validation. We trained an RF model on the training set of all factors.

**Gradient Boosting Decision Trees** (**GBDT)**

GBDT is a machine learning technique used in regression and classification tasks. It gives a prediction model in the form of an ensemble of weak prediction models, which are typically decision trees. It uses the gradient boosting method to train the trees. The algorithm was deployed in our data set with 14 trees in the forest, and the maximum depth of each tree was 4. These hyperparameters are also optimized through grid search and ten-fold cross-validation. We trained a GBDT model on the training set of all factors.

**Extreme Gradient Boosting Trees (XGBT)**

Both XGBT and GBDT follow the principle of gradient boosting. XGBT is a more regularized form of GBDT. XGBT uses advanced regularization (L1&L2), which improves model generalization capabilities. XGBT delivers high performance compared to GBDT, whose training is very fast and can be parallelized across clusters. The algorithm was deployed in our data set with a learning rate of 0.1, estimators of 26 and max depth of 4. We trained an XGBT model on the training set of all factors.

**Supplementary Table 1. Baseline characteristics between included and excluded patients.**

| **Characteristics** | **Included patients**  **(n=3514)** | **Excluded patients**  **(n=1538)** | ***P*** |
| --- | --- | --- | --- |
| **Demographics:** |  |  |  |
| Age, yrs | 66 (59, 74) | 67 (58, 73) | 0.198 |
| Female, n(%) | 1170 (33.3) | 486 (31.6) | 0.237 |
| Height, cm | 168 (160, 172) | 168 (160, 172) | 0.154 |
| Weight, kg | 70 (63, 77) | 70 (62, 76) | 0.052 |
| BMI, kg/m^2^ | 25.06 (23.39, 27.04) | 24.98 (22.99, 27.18) | 0.241 |
| **Medical history:** |  |  |  |
| Hypertension, n(%) | 2642 (75.2) | 1190 (77.4) | 0.094 |
| NYHA Classification, n(%) |  |  | 0.913 |
| NYHA Classification Ⅰ | 323 (9.2) | 136 (8.8) |  |
| NYHA Classification Ⅱ | 1631 (46.4) | 734 (47.7) |  |
| NYHA Classification Ⅲ | 418 (11.9) | 184 (12.0) |  |
| NYHA Classification Ⅳ | 71 (2.0) | 32 (2.1) |  |
| CHF, n(%) | 423 (12.4) | 184 (12.0) | 0.644 |
| Prior myocardial infarction, n(%) | 326 (9.3) | 133 (8.6) | 0.474 |
| Stable Angina, n(%) | 336 (9.6) | 129 (8.4) | 0.184 |
| ACS, n(%) | 1892 (53.8) | 830 (54.0) | 0.935 |
| Diabetes history, yrs |  |  | 0.900 |
| <1 | 170 (5.4) | 83 (5.4) |  |
| 1-5 | 602 (19.1) | 295 (19.2) |  |
| 5-10 | 859 (27.3) | 439 (28.5) |  |
| 10-20 | 1142 (36.3) | 550 (35.8) |  |
| >=20 | 371 (11.8) | 171 (11.1) |  |
| **CAG and PCI:** |  |  |  |
| Vessels of coronary artery disease, n(%) |  |  | <0.001 |
| 0 | 357 (10.2) | 36 (2.3) |  |
| 1 | 1021 (29.1) | 414 (27.0) |  |
| 2 | 1294 (36.8) | 532 (34.7) |  |
| 3 | 664 (18.9) | 457 (29.8) |  |
| 4 | 147 (4.2) | 79 (5.2) |  |
| 5 | 29 (0.8) | 12 (0.8) |  |
| 6 | 2 (0.1) | 2 (0.1) |  |
| Preoperative SBP, mmHg | 130 (123, 143) | 132 (121.5, 145.00) | 0.582 |
| Preoperative DBP, mmHg | 80 (70, 85) | 79 (70, 84) | 0.094 |
| **Contrast agent:** |  |  |  |
| Nonionic low-osmolar, n(%) | 1402 (39.9) | 622 (40.4) | 0.716 |
| Nonionic iso-osmolar, n(%) | 2060 (58.6) | 916 (59.6) | 0.534 |
| Volume of contrast agent, mL | 200 (100, 200) | 200 (100, 200) | 0.205 |

**Continued.**

| **Characteristics** | **Included patients**  **(n=3514)** | **Excluded patients**  **(n=1538)** | ***P*** |
| --- | --- | --- | --- |
| **Medications :** |  |  |  |
| Β-blocker, n(%) | 2137 (60.8) | 929 (60.4) | 0.783 |
| ACEI/ARB, n(%) | 2107 (60.0) | 878 (57.1) | 0.056 |
| Diuretics, n(%) | 885 (25.7) | 413 (26.9) | 0.409 |
| CCB, n(%) | 1001 (29.1) | 456 (29.6) | 0.689 |
| Insulins, n(%) | 1677 (47.7) | 728 (47.3) | 0.799 |
| Oral hypoglycemic agent, n(%) | 2072 (59.0) | 911 (59.2) | 0.858 |
| **Pre-procedural laboratory determinations:** |  |  |  |
| Glucose, mmol/L | 8.4 (6.68, 11.2) | 8.27 (6.45, 10.96) | 0.061 |
| BUN, mg/dL | 5.9 (4.88, 7.31) | 6.3 (5.17, 7.96) | <0.001 |
| Baseline creatinine, umol/L | 70 (59, 86) | 69 (58, 89.1) | 0.731 |
| eGFR, mL/min/1.73 m^2^ | 89.93 (74.39, 99.71) | 89.89 (71.07, 101.35) | 0.997 |
| CKD stage, n(%) |  |  | <0.001 |
| Stage 1 | 1749 (49.8) | 761 (49.5) |  |
| Stage 2 | 1289 (36.7) | 507 (33.0) |  |
| Stage 3 | 433 (12.6) | 185 (12.0) |  |
| Stage 4 | 33 (0.9) | 36 (2.3) |  |
| Stage 5 | 0 (0.0) | 48 (3.1) |  |
| Hemoglobin, g/L | 133 (122, 143) | 133 (121, 143) | 0.489 |
| Albumin, g/L | 39.1 (36.9, 41.6) | 39.3 (37, 41.5) | 0.681 |
| Uric acid, umol/L | 320 (258, 393) | 319.5 (254, 400) | 0.873 |
| Total cholesterol, mmol/L | 3.76 (3.07, 4.60) | 3.8 (3.14, 4.51) | 0.779 |
| Triglycerides, mmol/L | 1.44 (1.04, 2.07) | 1.46 (1.06, 2.00) | 0.611 |
| HDL, mmol/L | 0.96 (0.83, 1.14) | 0.98 (0.83, 1.15) | 0.567 |
| LDL, mmol/L | 2.13 (1.59, 2.83) | 2.16 (1.53, 2.73) | 0.055 |
| Urine protein level, n(%) |  |  | 0.557 |
| 0 | 2999 (85.3) | 1270 (85.3) |  |
| 1 | 319 (9.1) | 130 (8.7) |  |
| 2 | 144 (4.1) | 58 (3.9) |  |
| 3 | 52 (1.5) | 30 (2.0) |  |
| LVEF, % | 62 (55, 65) | 63 (56, 65) | 0.456 |

Abbreviations: CIAKI, contrast-induced acute kidney injury; BMI, body mass index; CKD, chronic kidney disease; CHF, congestive heart failure; ACS, acute coronary syndrome; SBP, systolic blood pressure; DBP, diastolic blood pressure; CCB, calcium channel blocker; ACEI, angiotensin-converting enzyme inhibitor; ARB, angiotensin receptor blocker; eGFR, estimated glomerular filtration rate; HDL, high-density lipoprotein; LDL, low-density lipoprotein; LVEF, left ventricular ejection fraction.

**Supplementary Table 2. Baseline characteristics of patients in the three groups.**

| **Variables** | **All (N=3514)** | **Training set (N=2368)** | **Internal validation set (N=592)** | **External validation set (N=554)** | ***P*** |
| --- | --- | --- | --- | --- | --- |
| **Demographics** | | | | | |
| Age, yr | 66(59, 74) | 67(60, 74) | 67(59, 73) | 65(58, 73) | 0.007 |
| Female, n(%) | 1170(33.3) | 748(31.6) | 203(34.3) | 219(39.5) | 0.001 |
| Weight, kg | 70(63, 77) | 70(63, 77) | 70(62, 78) | 70(63, 75.4) | 0.518 |
| Height, cm | 168(160, 172) | 168(160, 172) | 167(160, 172) | 168(160, 173) | 0.502 |
| BMI, kg/m^2^ | 25.1(23.4, 27) | 25.1(23.4, 27.1) | 25.1(23, 27.3) | 25.1(23, 27.3) | 0.144 |
| **Medical history, n(%)** | | | | | |
| Anemia | 1074(30.6) | 777(32.8) | 191(32.3) | 106(19.1) | <0.001 |
| Hypertension | 2642(75.2) | 1829(77.2) | 444(75) | 369(66.6) | <0.001 |
| CKD | 475(13.5) | 336(14.2) | 83(14) | 56(10.1) | 0.038 |
| Prior myocardial infarction | 326(9.3) | 246(10.4) | 43(7.3) | 37(6.7) | 0.005 |
| Stable Angina | 336(9.6) | 252(10.6) | 52(8.8) | 32(5.8) | 0.002 |
| ACS | 1892(53.8) | 1159(48.9) | 280(47.3) | 453(81.8) | <0.001 |
| CHF | 423(12.4) | 284(12.4) | 71(12.5) | 68(12.5) | 0.937 |
| NYHA Classification, n(%) |  |  |  |  |  |
| NYHA Classification Ⅰ | 323(13.2) | 125(7.6) | 39(9.5) | 159(41.6) | <0.001 |
| NYHA Classification Ⅱ | 1631(66.8) | 1188(72.0) | 287(70.0) | 156(40.8) |
| NYHA Classification Ⅲ | 418(17.1) | 295(17.9) | 68(16.6) | 55(14.4) |
| NYHA Classification Ⅳ | 71(2.9) | 43(2.6) | 16(3.9) | 12(3.1) |
| Diabetes history, yrs |  |  |  |  |  |
| <1 | 170(5.4) | 88(4.2) | 27(5.2) | 55(9.9) | <0.001 |
| 1-5 | 605(19.2) | 361(17.4) | 77(14.8) | 167(30.1) |
| 5-10 | 865(27.4) | 572(27.5) | 173(33.1) | 120(21.7) |
| 10-20 | 1143(36.2) | 813(39.1) | 183(35.1) | 147(26.5) |
| >=20 | 372(11.8) | 245(11.8) | 62(11.9) | 65(11.7) |
| **CAG and PCI** | | | | | |
| Single-vessel disease, n(%) | 1021(29.1) | 741(31.3) | 196(33.1) | 84(15.2) | <0.001 |
| Multi-vessel disease, n(%) | 2136(60.8) | 1361(57.5) | 328(55.4) | 447(80.7) | <0.001 |
| Vessels of coronary artery disease, n(%) |  |  |  |  |  |
| 0 | 357(10.2) | 266(11.2) | 68(11.5) | 23(4.2) | <0.001 |
| 1 | 1021(29.1) | 741(31.3) | 196(33.1) | 84(15.2) |
| 2 | 1294(36.8) | 911(38.5) | 225(38) | 158(28.5) |
| 3 | 664(18.9) | 360(15.2) | 76(12.8) | 228(41.2) |
| 4 | 147(4.2) | 74(3.1) | 23(3.9) | 50(9) |
| 5 | 29(0.8) | 14(0.6) | 4(0.7) | 11(2) |
| 6 | 2(0.1) | 2(0.1) | 0(0) | 0(0) |
| Preoperative SBP, mmHg | 130(123, 143) | 130(122, 142) | 130(120, 140.8) | 130(120, 140.8) | 0.005 |

**Continued.**

| **Variables** | **All (N=3514)** | **Training set (N=2368)** | **Internal validation set (N=592)** | **External validation set (N=554)** | ***P*** |
| --- | --- | --- | --- | --- | --- |
| Preoperative DBP, mmHg | 80(70, 85) | 80(70, 84) | 80(70, 83) | 80(70, 87.3) | 0.309 |
| **Contrast agent** | | | | | |
| Nonionic low-osmolar, n(%) | 1402(39.9) | 828(35) | 210(35.5) | 364(65.7) | <0.001 |
| Nonionic iso-osmolar, n(%) | 2060(58.6) | 1488(62.8) | 363(61.3) | 209(37.7) | <0.001 |
| Volume of contrast agent, mL | 200(100, 200) | 200(100, 200) | 200(100, 200) | 150(100, 200) | <0.001 |
| **Medications, n(%)** |  |  |  |  |  |
| Β-blocker | 2137(60.8) | 1427(60.3) | 351(59.3) | 359(64.8) | 0.101 |
| ACEI/ARB | 2107(60) | 1393(58.8) | 354(59.8) | 360(65) | 0.029 |
| Diuretics | 885(25.7) | 508(22) | 126(21.8) | 251(45.3) | <0.001 |
| CCB | 1001(29.1) | 638(27.6) | 154(26.7) | 209(37.7) | <0.001 |
| Insulins | 1677(47.7) | 1211(51.1) | 287(48.5) | 179(32.3) | <0.001 |
| Oral hypoglycemic agent | 2072(59) | 1362(57.5) | 309(52.2) | 401(72.4) | <0.001 |
| **Pre-procedural laboratory determinations** | | | | | |
| Glucose, mmol/L | 8.4(6.7, 11.2) | 8.4(6.7, 11.3) | 8.6(6.9, 11.4) | 8.6(6.9, 11.4) | 0.073 |
| Hemoglobin, g/L | 133(122, 143) | 133(122, 143) | 132(122, 143) | 134(124, 145) | 0.023 |
| Albumin, g/L | 39.1(36.9, 41.6) | 38.7(36.6, 41) | 38.7(36.5, 40.9) | 38.7(36.5, 40.9) | <0.001 |
| Uric acid, umol/L | 320(258, 393) | 320(258, 396.5) | 326(260, 401.5) | 312(256.8, 374) | 0.053 |
| Total cholesterol, mmol/L | 3.8(3.1, 4.6) | 3.7(3, 4.5) | 3.8(3.1, 4.6) | 3.8(3.1, 4.6) | <0.001 |
| Triglycerides, mmol/L | 1.5(1.1, 2.1) | 1.4(1, 2) | 1.5(1.1, 2.1) | 1.5(1.1, 2.1) | <0.001 |
| HDL, mmol/L | 1(0.8, 1.1) | 0.9(0.8, 1.1) | 0.9(0.8, 1.1) | 0.9(0.8, 1.1) | <0.001 |
| LDL, mmol/L | 2.1(1.6, 2.8) | 2.1(1.6, 2.8) | 2.1(1.6, 2.8) | 2.1(1.6, 2.8) | <0.001 |
| BUN, mg/dL | 5.9(4.9, 7.3） | 6(4.9, 7.3) | 5.9(4.8, 7.5) | 5.9(4.8, 7.5) | 0.055 |
| Baseline creatinine, umol/L | 70(59, 86) | 71(60, 87) | 71(61, 87.2) | 71(61, 87.2) | <0.001 |
| eGFR, mL/min/1.73 m^2^ | 90(74.4, 99.7) | 89.4(73.2, 98.4) | 88.8(71.9, 99) | 88.8(71.9, 99) | <0.001 |
| CKD |  |  |  |  |  |
| stage 1 | 1749(49.8) | 1136(48) | 280(47.3) | 333(60.1) | <0.001 |
| stage 2 | 1289(36.7) | 895(37.8) | 229(38.7) | 165(29.8) |
| stage 3 | 443(12.6) | 314(13.3) | 80(13.5) | 49(8.8) |
| stage 4 | 33(0.9) | 23(1) | 3(0.5) | 7(1.3) |
| Proteinuria, n(%) | 506(14.4) | 293(12.4) | 72(12.2) | 141(25.5) | <0.001 |
| Urine protein level, n(%) |  |  |  |  |  |
| 0 | 2999(85.3) | 2075(87.6) | 520(87.8) | 404(72.9) | <0.001 |
| 1 | 319(9.1) | 177(7.5) | 50(8.4) | 92(16.6) |
| 2 | 144(4.1) | 83(3.5) | 16(2.7) | 45(8.1) |
| 3 | 52(1.5) | 33(1.4) | 6(1) | 13(2.3) |
| LVEF, % | 62(55, 65) | 63(56, 65) | 63(56, 65) | 58(52, 62) | <0.001 |

Abbreviations: CIAKI, contrast-induced acute kidney injury; BMI, body mass index; CKD, chronic kidney disease; ACS, acute coronary syndrome; CHF, congestive heart failure; SBP, systolic blood pressure; DBP, diastolic blood pressure; CCB, calcium channel blocker; ACEI, angiotensin-converting enzyme inhibitor; ARB, angiotensin receptor blocker; eGFR, estimated glomerular filtration rate; HDL, high-density lipoprotein; LDL, low-density lipoprotein; LVEF, left ventricular ejection fraction.

**Supplementary table 3. Ten-fold cross-validation results of AUC and accuracy in models.**

| **Classifier** | **AUC(%)** (IQR) | **Accuracy(%)** (IQR) |
| --- | --- | --- |
| Mehran score | 65.97(64.17, 68.41) | 80.31(79.63, 80.35) |
| XGBT | 80.09(75.58, 82.27) | 81.62(80.26, 83.62) |
| RF | 80.01(76.76, 82.14) | 80.57(79.71, 81.71) |
| GBDT | 80.10(72.35, 81.62) | 80.79(80.61, 81.77) |
| SVM | 78.42(74.34, 80.78) | 70.18(67.25, 71.69) |
| LASSO+LR | 78.57(75.47, 81.69) | 81.84(79.28, 83.30) |
| LR | 77.39(75.51, 81.13) | 81.62(79.71, 82.64) |

Abbreviations: LR, logistic regression; LASSO, least absolute shrinkage and selection operator; SVM, support vector machine; GBDT, gradient boosted decision trees; XGBT, extreme gradient boosting trees; RF, random forest; AUC, area under the curve. Variables are presented as medians (interquartile ranges [IQR]).

**Supplementary Table 4. Model performance using different balancing methods.**

| **Balance** | **Model** | **AUC (%)**  **(95% CI)** | **Accuracy (%)**  **(95% CI)** | **Sensitivity (%)**  **(95% CI)** | **Specificity (%)**  **(95% CI)** | **PPV (%)**  **(95% CI)** | **NPV (%)**  **(95% CI)** | ***F_1_* score (%)**  **(95% CI)** |
| --- | --- | --- | --- | --- | --- | --- | --- | --- |
| **Internal validation performance** | | | | | | | | |
| Before balancing | LR | 79.87  (75.79-83.28) | 78.04  (75.17-80.91) | 56.07  (47.79-64.44) | 82.89  (79.96-85.77) | 41.96  (35.29-48.61) | 89.53  (87.05-91.92) | 48.00  (41.15-54.18) |
|  | LASSO+LR | 79.01  (74.68-82.69) | 74.83  (71.96-77.70) | 63.55  (55.67-71.30) | 77.32  (74.16-80.29) | 38.20  (32.28-43.96) | 90.58  (88.21-92.94) | 47.72  (41.45-53.29) |
|  | SVM | 80.68  (76.66-84.04) | 77.70  (74.83-80.41) | 57.94  (50.00-66.04) | 82.06  (79.18-84.84) | 41.61  (34.81-47.97) | 89.84  (87.50-92.26) | 48.44  (42.11-54.62) |
|  | RF | 78.94  (75.16-82.60) | 78.38  (75.51-81.08) | 56.07  (48.46-63.64) | 83.30  (80.53-86.12) | 42.55  (35.67-49.30) | 89.58  (87.16-91.92) | 48.39  (41.67-54.41) |
|  | GBDT | 82.83  (79.12-86.14) | 82.43  (79.73-84.97) | 56.07  (48.28-63.48) | 88.25  (85.59-90.47) | 51.28  (43.61-58.72) | 90.11  (87.76-92.28) | 53.57  (46.49-59.91) |
|  | XGBT | 83.19  (79.35-86.64) | 82.94  (80.57-85.47) | 62.62  (55.17-70.19) | 87.42  (84.91-89.76) | 52.34  (45.14-59.29) | 91.38  (89.22-93.53) | 57.02  (50.22-63.11) |
| TomekLinks | LR | 79.90  (75.95 - 83.32) | 78.21  (75.34 - 81.08) | 57.01  (49.04 - 64.55) | 82.89  (80.00 - 85.66) | 42.36  (35.62 - 48.82) | 89.73  (87.39 - 92.01) | 48.61  (41.92 - 54.62) |
|  | LASSO+LR | 79.92  (75.91 - 83.44) | 78.21  (75.68 - 80.74) | 56.07  (47.97 - 63.46) | 83.09  (80.21 - 85.71) | 42.25  (35.17 - 48.63) | 89.56  (87.17 - 91.96) | 48.19  (41.30 - 54.10) |
|  | SVM | 76.88  (72.46 - 80.68) | 76.35  (73.65 - 79.05) | 53.27  (45.54 - 61.47) | 81.44  (78.50 - 84.26) | 38.78  (32.31 - 45.06) | 88.76  (86.35 - 91.28) | 44.88  (38.30 - 51.03) |
|  | RF | 79.76  (76.07 - 83.06) | 78.89  (76.18 - 81.59) | 53.27  (45.61 - 61.11) | 84.54  (81.78 - 86.95) | 43.18  (36.17 - 50.00) | 89.13  (86.75 - 91.54) | 47.70  (40.98 - 53.85) |
|  | GBDT | 80.28  (76.49 - 83.98) | 80.41  (77.70 - 83.11) | 57.01  (49.51 - 64.81) | 85.57  (82.85 - 88.02) | 46.56  (39.31 - 53.78) | 90.02  (87.78 - 92.36) | 51.26  (44.64 - 57.52) |
|  | XGBT | 81.59  (77.71 - 85.33) | 81.08  (78.55 - 83.78) | 54.21  (46.23 - 62.26) | 87.01  (84.66 - 89.41) | 47.93  (40.59 - 55.36) | 89.60  (87.32 - 91.97) | 50.88  (44.05 - 57.26) |
| SMOTE | LR | 79.71  (75.36 - 83.15) | 63.51  (60.14 - 66.55) | 85.05  (79.05 - 90.48) | 58.76  (54.94 - 62.24) | 31.27  (26.89 - 35.46) | 94.68  (92.33 - 96.75) | 45.73  (40.49 - 50.49) |
|  | LASSO+LR | 79.44  (75.19 - 83.11) | 65.71  (62.67 - 68.75) | 83.18  (76.98 - 88.78) | 61.86  (58.39 - 65.19) | 32.48  (27.90 - 37.06) | 94.34  (92.14 - 96.37) | 46.72  (41.29 - 51.83) |

**Continued.**

| **Balance** | **Model** | **AUC (%)**  **(95% CI)** | **Accuracy (%)**  **(95% CI)** | **Sensitivity (%)**  **(95% CI)** | **Specificity (%)**  **(95% CI)** | **PPV (%)**  **(95% CI)** | **NPV (%)**  **(95% CI)** | ***F_1_* score (%)**  **(95% CI)** |
| --- | --- | --- | --- | --- | --- | --- | --- | --- |
| SMOTE | SVM | 79.45  (75.16 - 82.93) | 60.30  (56.93 - 63.34) | 87.85  (82.46 - 92.73) | 54.23  (50.50 - 57.55) | 29.75  (25.57 - 33.85) | 95.29  (93.13 - 97.32) | 44.44  (39.24 - 49.07) |
|  | RF | 78.31  (74.54 - 81.81) | 77.53  (74.83 - 80.41) | 55.14  (47.06 - 63.11) | 82.47  (79.70 - 85.29) | 40.97  (34.44 - 47.69) | 89.29  (86.76 - 91.72) | 47.01  (40.34 - 53.18) |
|  | GBDT | 82.20  (78.54 - 85.63) | 81.42  (78.89 - 83.95) | 51.40  (43.12 - 59.79) | 88.04  (85.74 - 90.31) | 48.67  (40.91 - 56.03) | 89.14  (86.90 - 91.53) | 50.00  (42.79 - 56.48) |
|  | XGBT | 82.57  (78.91 - 85.81) | 81.76  (79.05 - 84.29) | 50.47  (42.34 - 58.76) | 88.66  (86.21 - 90.82) | 49.54  (40.98 - 57.27) | 89.03  (86.72 - 91.49) | 50.00  (42.53 - 56.74) |
| ADASYN | LR | 80.02  (75.92 - 83.37) | 60.98  (57.60 - 64.02) | 89.72  (84.62 - 94.23) | 54.64  (51.01 - 58.04) | 30.38  (26.21 - 34.41) | 96.01  (93.94 - 97.83) | 45.39  (40.28 - 50.00) |
|  | LASSO+LR | 79.83  (75.70 - 83.41) | 57.09  (53.89 - 60.14) | 90.65  (85.87 - 95.12) | 49.69  (46.09 - 53.12) | 28.45  (24.56 - 32.39) | 96.02  (93.70 - 97.99) | 43.30  (38.36 - 47.75) |
|  | SVM | 78.04  (73.90 - 81.86) | 58.61  (55.24 - 61.82) | 88.79  (83.65 - 93.62) | 51.96  (48.31 - 55.47) | 28.96  (24.85 - 32.82) | 95.45  (92.97 - 97.52) | 43.68  (38.53 - 48.05) |
|  | RF | 77.49  (73.52 - 81.07) | 76.35  (73.65 - 79.22) | 52.34  (44.44 - 60.36) | 81.65  (78.85 - 84.47) | 38.62  (32.14 - 45.52) | 88.59  (86.06 - 91.10) | 44.44  (37.60 - 50.79) |
|  | GBDT | 81.69  (78.00 - 84.92) | 80.41  (77.70 - 83.11) | 52.34  (44.23 - 60.20) | 86.60  (84.02 - 88.93) | 46.28  (38.39 - 53.73) | 89.17  (86.80 - 91.58) | 49.12  (42.11 - 55.77) |
|  | XGBT | 81.13  (77.23 - 84.44) | 82.77  (80.07 - 85.30) | 47.66  (39.80 - 55.56) | 90.52  (88.26 - 92.63) | 52.58  (43.81 - 60.61) | 88.69  (86.33 - 91.06) | 50.00  (42.16 - 56.73) |
| Random  Oversampling | LR | 80.28  (76.24 - 83.84) | 61.82  (58.45 - 64.86) | 88.79  (83.84 - 93.86) | 55.88  (51.93 - 59.44) | 30.74  (26.44 - 35.07) | 95.76  (93.77 - 97.64) | 45.67  (40.48 - 50.59) |
|  | LASSO+LR | 79.64  (75.56 - 83.20) | 62.16  (58.78 - 65.20) | 85.05  (79.25 - 90.48) | 57.11  (53.44 - 60.73) | 30.43  (25.89 - 34.84) | 94.54  (92.28 - 96.58) | 44.83  (39.45 - 49.57) |
|  | SVM | 80.58  (76.65 - 83.94) | 63.51  (60.14 - 66.72) | 89.72  (84.75 - 94.34) | 57.73  (53.96 - 61.28) | 31.89  (27.57 - 36.25) | 96.22  (94.29 - 97.97) | 47.06  (41.81 - 51.85) |
|  | RF | 80.69  (76.71 - 84.33) | 76.69  (73.99 - 79.39) | 71.96  (64.41 - 78.51) | 77.73  (74.64 - 80.57) | 41.62  (35.83 - 47.25) | 92.63  (90.48 - 94.72) | 52.74  (46.85 - 58.11) |

**Continued.**

| **Balance** | **Model** | **AUC (%)**  **(95% CI)** | **Accuracy (%)**  **(95% CI)** | **Sensitivity (%)**  **(95% CI)** | **Specificity (%)**  **(95% CI)** | **PPV (%)**  **(95% CI)** | **NPV (%)**  **(95% CI)** | ***F_1_* score (%)**  **(95% CI)** |
| --- | --- | --- | --- | --- | --- | --- | --- | --- |
| Random  Oversampling | GBDT | 81.02  (77.12 - 84.58) | 79.56  (77.03 - 82.26) | 60.75  (52.29 - 68.09) | 83.71  (81.22 - 86.37) | 45.14  (38.41 - 52.08) | 90.62  (88.30 - 92.94) | 51.79  (44.84 - 57.83) |
|  | XGBT | 81.72  (77.92 - 85.15) | 77.70  (75.00 - 80.57) | 67.29  (59.66 - 74.53) | 80.00  (77.16 - 82.90) | 42.60  (36.36 - 48.62) | 91.73  (89.51 - 93.81) | 52.17  (45.80 - 57.82) |
| Random  Undersampling | LR | 80.45  (76.70 - 83.71) | 64.19  (60.81 - 67.23) | 85.98  (80.41 - 91.15) | 59.38  (55.73 - 62.92) | 31.83  (27.34 - 36.24) | 95.05  (92.99 - 96.99) | 46.46  (41.29 - 51.43) |
|  | LASSO+LR | 79.59  (75.51 - 82.88) | 59.12  (55.74 - 62.50) | 86.92  (81.32 - 92.17) | 52.99  (49.18 - 56.58) | 28.97  (24.84 - 32.93) | 94.83  (92.51 - 97.00) | 43.46  (38.21 - 48.17) |
|  | SVM | 80.41  (76.67 - 83.87) | 64.86  (61.49 - 68.07) | 84.11  (78.10 - 89.52) | 60.62  (56.68 - 64.05) | 32.03  (27.48 - 36.43) | 94.53  (92.28 - 96.47) | 46.39  (41.05 - 51.27) |
|  | RF | 80.02  (76.45 - 83.37) | 73.65  (70.78 - 76.52) | 71.03  (63.46 - 77.88) | 74.23  (70.85 - 77.28) | 37.81  (32.14 - 43.30) | 92.07  (89.83 - 94.24) | 49.35  (43.39 - 54.89) |
|  | GBDT | 80.25  (76.64 - 83.76) | 70.61  (67.57 - 73.48) | 76.64  (70.10 - 83.00) | 69.28  (65.92 - 72.64) | 35.50  (30.40 - 40.43) | 93.07  (90.86 - 95.30) | 48.52  (42.95 - 53.61) |
|  | XGBT | 81.54  (77.95 - 84.78) | 71.62  (68.58 - 74.66) | 72.90  (66.00 - 79.59) | 71.34  (67.86 - 74.79) | 35.94  (30.56 - 41.15) | 92.27  (90.00 - 94.52) | 48.15  (42.32 - 53.25) |
| **External validation performance** | | | | | | | | |
| Before balancing | LR | 77.82  (72.91-82.71) | 72.02  (68.95-75.27) | 71.25  (62.79-79.27) | 72.15  (68.91-75.59) | 30.16  (25.00-35.71) | 93.70  (91.44-95.66) | 42.38  (36.05-48.44) |
|  | LASSO+LR | 76.10  (70.68-81.13) | 67.15  (63.90-70.22) | 73.75  (65.33-82.05) | 66.03  (62.53-69.57) | 26.82  (22.17-31.80) | 93.71  (91.36-95.92) | 39.33  (33.68-45.18) |
|  | SVM | 77.41  (72.27-82.18) | 67.87  (64.62-71.12) | 71.25  (62.82-79.27) | 67.30  (63.83-70.82) | 26.89  (22.06-32.00) | 93.27  (90.88-95.30) | 39.04  (32.98-44.84) |
|  | RF | 76.89  (71.76-81.85) | 72.38  (69.49-75.63) | 70.00  (61.54-78.65) | 72.78  (69.62-76.15) | 30.27  (25.25-35.93) | 93.50  (91.36-95.51) | 42.26  (36.15-48.41) |
|  | GBDT | 77.12  (72.32-81.64) | 74.37  (71.48-77.44) | 63.75  (54.79-72.37) | 76.16  (72.98-79.32) | 31.10  (25.47-37.18) | 92.56  (90.26-94.55) | 41.80  (35.19-48.15) |

**Continued.**

| **Balance** | **Model** | **AUC (%)**  **(95% CI)** | **Accuracy (%)**  **(95% CI)** | **Sensitivity (%)**  **(95% CI)** | **Specificity (%)**  **(95% CI)** | **PPV (%)**  **(95% CI)** | **NPV (%)**  **(95% CI)** | ***F_1_* score (%)**  **(95% CI)** |
| --- | --- | --- | --- | --- | --- | --- | --- | --- |
| Before balancing | XGBT | 78.10  (72.95-82.70) | 72.92  (69.86-75.99) | 71.25  (62.79-79.73) | 73.21  (69.89-76.34) | 30.98  (25.63-36.55) | 93.78  (91.62-95.79) | 43.18  (36.89-49.31) |
| TomekLinks | LR | 76.94  (71.84 - 82.03) | 72.02  (68.95 - 75.09) | 71.25  (62.82 - 79.22) | 72.15  (68.92 - 75.48) | 30.16  (25.00 - 35.67) | 93.70  (91.42 - 95.71) | 42.38  (36.36 - 48.63) |
|  | LASSO+LR | 79.31  (74.40 - 84.17) | 72.20  (69.13 - 75.27) | 73.75  (65.17 - 82.05) | 71.94  (68.78 - 75.26) | 30.73  (25.77 - 36.04) | 94.20  (91.98 - 96.21) | 43.38  (37.36 - 49.64) |
|  | SVM | 71.64  (65.86 - 76.92) | 72.74  (69.68 - 75.81) | 56.25  (46.25 - 64.94) | 75.53  (72.27 - 78.89) | 27.95  (22.37 - 33.53) | 91.09  (88.61 - 93.33) | 37.34  (30.51 - 43.62) |
|  | RF | 80.10  (75.33 - 84.64) | 72.56  (69.49 - 75.63) | 75.00  (66.67 - 82.89) | 72.15  (68.90 - 75.48) | 31.25  (26.02 - 36.95) | 94.48  (92.39 - 96.46) | 44.12  (38.02 - 50.52) |
|  | GBDT | 78.16  (73.27 - 83.01) | 72.38  (69.31 - 75.27) | 76.25  (68.24 - 83.75) | 71.73  (68.39 - 74.95) | 31.28  (26.09 - 36.59) | 94.71  (92.76 - 96.47) | 44.36  (38.38 - 50.49) |
|  | XGBT | 81.65  (77.04 - 86.09) | 75.99  (73.10 - 79.06) | 72.50  (64.00 - 80.82) | 76.58  (73.24 - 79.74) | 34.32  (28.57 - 40.34) | 94.29  (92.19 - 96.16) | 46.59  (40.17 - 52.80) |
| SMOTE | LR | 72.94  (67.40 - 78.41) | 40.43  (37.00 - 43.68) | 87.50  (80.88 - 93.18) | 32.49  (28.94 - 35.80) | 17.95  (15.00 - 21.11) | 93.90  (90.57 - 96.73) | 29.79  (25.44 - 34.14) |
|  | LASSO+LR | 75.70  (70.29 - 80.95) | 44.77  (41.52 - 48.38) | 83.75  (76.67 - 90.28) | 38.19  (34.73 - 41.70) | 18.61  (15.51 - 21.86) | 93.30  (90.05 - 96.20) | 30.45  (25.99 - 34.76) |
|  | SVM | 72.07  (66.45 - 77.76) | 32.13  (29.06 - 35.38) | 88.75  (82.72 - 94.38) | 22.57  (19.51 - 25.43) | 16.21  (13.53 - 19.17) | 92.24  (88.03 - 96.26) | 27.41  (23.39 - 31.64) |
|  | RF | 70.23  (64.67 - 75.70) | 63.54  (60.29 - 66.97) | 66.25  (57.33 - 74.42) | 63.08  (59.45 - 66.74) | 23.25  (18.78 - 27.80) | 91.72  (89.06 - 94.07) | 34.42  (28.78 - 39.87) |
|  | GBDT | 73.28  (68.51 - 78.12) | 71.84  (68.77 - 74.91) | 58.75  (49.43 - 67.95) | 74.05  (70.82 - 77.39) | 27.65  (22.16 - 33.33) | 91.41  (89.12 - 93.61) | 37.60  (31.18 - 44.02) |
|  | XGBT | 72.15  (67.36 - 76.86) | 67.15  (63.90 - 70.40) | 55.00  (45.35 - 64.00) | 69.20  (65.69 - 72.73) | 23.16  (18.03 - 28.02) | 90.11  (87.37 - 92.43) | 32.59  (25.98 - 38.36) |
| ADASYN | LR | 73.05  (67.70 - 78.47) | 33.75  (30.69 - 36.82) | 88.75  (82.28 - 94.12) | 24.47  (21.44 - 27.60) | 16.55  (13.83 - 19.50) | 92.80  (88.70 - 96.26) | 27.90  (23.86 - 32.05) |

**Continued.**

| **Balance** | **Model** | **AUC (%)**  **(95% CI)** | **Accuracy (%)**  **(95% CI)** | **Sensitivity (%)**  **(95% CI)** | **Specificity (%)**  **(95% CI)** | **PPV (%)**  **(95% CI)** | **NPV (%)**  **(95% CI)** | ***F_1_* score (%)**  **(95% CI)** |
| --- | --- | --- | --- | --- | --- | --- | --- | --- |
| ADASYN | LASSO+LR | 75.40  (69.96 - 80.71) | 29.96  (26.90 - 33.03) | 92.50  (87.50 - 97.22) | 19.41  (16.42 - 22.34) | 16.23  (13.55 - 19.04) | 93.88  (89.69 - 97.78) | 27.61  (23.66 - 31.64) |
|  | SVM | 71.85  (66.11 - 77.60) | 27.08  (24.01 - 30.14) | 91.25  (85.92 - 96.20) | 16.24  (13.45 - 19.00) | 15.53  (12.88 - 18.30) | 91.67  (86.75 - 96.39) | 26.55  (22.52 - 30.63) |
|  | RF | 70.02  (64.63 - 75.33) | 66.06  (62.82 - 69.49) | 58.75  (50.00 - 67.37) | 67.30  (63.83 - 70.93) | 23.27  (18.56 - 28.21) | 90.62  (87.90 - 93.02) | 33.33  (27.44 - 39.19) |
|  | GBDT | 71.73  (67.24 - 76.19) | 70.94  (67.87 - 74.01) | 50.00  (40.51 - 59.02) | 74.47  (71.28 - 77.78) | 24.84  (19.64 - 30.67) | 89.82  (87.27 - 92.25) | 33.20  (26.79 - 39.36) |
|  | XGBT | 74.10  (69.44 - 78.77) | 76.17  (73.29 - 79.24) | 51.25  (41.98 - 60.24) | 80.38  (77.45 - 83.54) | 30.60  (24.31 - 37.59) | 90.71  (88.50 - 92.87) | 38.32  (31.07 - 44.90) |
| Random  Oversampling | LR | 79.11  (74.10 - 83.87) | 38.99  (35.92 - 42.24) | 91.25  (85.71 - 96.05) | 30.17  (26.79 - 33.55) | 18.07  (15.20 - 21.18) | 95.33  (92.26 - 97.96) | 30.17  (25.96 - 34.36) |
|  | LASSO+LR | 80.04  (75.02 - 84.89) | 41.52  (38.27 - 44.95) | 90.00  (84.06 - 95.18) | 33.33  (29.85 - 36.82) | 18.56  (15.52 - 21.88) | 95.18  (92.22 - 97.75) | 30.77  (26.38 - 35.27) |
|  | SVM | 79.02  (74.07 - 83.72) | 37.00  (33.94 - 40.25) | 90.00  (83.82 - 95.12) | 28.06  (24.79 - 31.41) | 17.43  (14.50 - 20.54) | 94.33  (90.91 - 97.16) | 29.21  (24.89 - 33.40) |
|  | RF | 75.82  (70.78 - 81.00) | 57.22  (53.97 - 60.65) | 85.00  (78.26 - 91.18) | 52.53  (48.93 - 56.30) | 23.21  (19.43 - 27.27) | 95.40  (93.16 - 97.38) | 36.46  (31.44 - 41.67) |
|  | GBDT | 75.48  (70.84 - 80.22) | 74.01  (70.94 - 76.90) | 58.75  (50.00 - 68.06) | 76.58  (73.42 - 79.62) | 29.75  (24.14 - 35.62) | 91.67  (89.28 - 93.81) | 39.50  (33.18 - 45.67) |
|  | XGBT | 75.18  (70.05 - 80.09) | 71.66  (68.41 - 74.73) | 70.00  (61.04 - 78.21) | 71.94  (68.50 - 75.31) | 29.63  (24.32 - 35.00) | 93.42  (91.13 - 95.48) | 41.64  (35.38 - 47.69) |
| Random  Undersampling | LR | 74.01  (68.56 - 79.29) | 38.99  (35.74 - 42.24) | 91.25  (85.88 - 96.15) | 30.17  (26.99 - 33.48) | 18.07  (15.25 - 21.21) | 95.33  (92.31 - 97.99) | 30.17  (26.00 - 34.59) |
|  | LASSO+LR | 78.10  (73.19 - 82.76) | 33.39  (30.14 - 36.64) | 93.75  (89.04 - 97.73) | 23.21  (20.17 - 26.41) | 17.08  (14.32 - 20.09) | 95.65  (92.37 - 98.45) | 28.90  (24.80 - 33.09) |
|  | SVM | 76.58  (71.43 - 81.41) | 41.52  (38.27 - 44.95) | 88.75  (82.67 - 94.05) | 33.54  (30.17 - 37.00) | 18.39  (15.40 - 21.69) | 94.64  (91.61 - 97.26) | 30.47  (26.22 - 34.97) |

**Continued**

Abbreviations: LR, logistic regression; LASSO, least absolute shrinkage and selection operator; SVM, support vector machine; RF, random forest; GBDT, gradient boosted decision trees; XGBT, extreme gradient boosting trees; AUC, area under the curve; PPV, positive predictive value; NPV, negative predictive value; 95% CI, 95% confidence interval; SMOTE, synthetic minority oversampling technique; ADASYN, adaptive synthetic technique.

| **Balance** | **Model** | **AUC (%)**  **(95% CI)** | **Accuracy (%)**  **(95% CI)** | **Sensitivity (%)**  **(95% CI)** | **Specificity (%)**  **(95% CI)** | **PPV (%)**  **(95% CI)** | **NPV (%)**  **(95% CI)** | ***F_1_* score (%)**  **(95% CI)** |
| --- | --- | --- | --- | --- | --- | --- | --- | --- |
| Random  Undersampling | RF | 76.93  (72.14 - 81.64) | 48.74  (45.49 - 52.35) | 85.00  (78.41 - 91.46) | 42.62  (39.03 - 46.40) | 20.00  (16.62 - 23.62) | 94.39  (91.71 - 96.79) | 32.38  (27.75 - 37.26) |
|  | GBDT | 72.70  (67.43 - 77.39) | 44.58  (41.16 - 48.19) | 87.50  (81.18 - 93.02) | 37.34  (33.82 - 41.05) | 19.07  (15.97 - 22.46) | 94.65  (91.63 - 97.06) | 31.32  (26.85 - 35.94) |
|  | XGBT | 72.91  (68.05 - 77.76) | 51.26  (48.01 - 54.69) | 83.75  (76.25 - 90.24) | 45.78  (42.06 - 49.35) | 20.68  (17.38 - 24.37) | 94.35  (91.59 - 96.64) | 33.17  (28.57 - 37.98) |

**Supplementary Table 5.**  **The median [IQR] of the continuous features in BCPMD.**

| Features | CIAKI (n=634) | Non-CIAKI (n=2880) | *P* value |
| --- | --- | --- | --- |
| Preoperative DBP (mmHg) | 80.00 (70.00, 90.00) | 80.00 (70.00, 84.00) | 0.012 |
| Glucose, mmol/L | 8.88 (7.09, 11.52) | 8.29 (6.60, 11.10) | 0.001 |
| Albumin (g/L) | 38.30 (35.20, 40.70) | 39.30 (37.20, 41.80) | <0.001 |
| Uric acid (umol/L) | 362.00 (289.00, 444.00) | 311.00 (254.25, 383.00) | <0.001 |
| LVEF (%) | 59.00 (45.00, 64.00) | 63.00 (56.33, 65.00) | <0.001 |
| Hemoglobin (g/L) | 129.00 (114.00, 141.00) | 133.00 (123.00, 144.00) | <0.001 |
| Baseline creatinine (umol/L) | 76.00 (62.00, 104.00) | 70.00 (58.75, 83.00) | <0.001 |
| Volume of contrast agent (mL) | 200.00 (170.00, 200.00) | 200.00 (100.00, 200.00) | <0.001 |

Abbreviations: CIAKI, contrast-induced acute kidney injury; DBP, diastolic blood pressure; LVEF, left ventricular ejection fraction; IQR, interquartile range; BCPMD, brief CIAKI prediction model for diabetes.

**Supplementary table 6. Baseline characteristics of patients in the prospective cohort.**

| **Characteristic** | **Non-CIAKI**  **(n=153)** | **CIAKI**  **(n=19)** | ***P*** |
| --- | --- | --- | --- |
| ACS, n(%) | 121 (79.1) | 17 (89.5) | 0.284 |
| Stable Angina, n(%) | 11 (7.2) | 0 (0.0) | 0.227 |
| CHF, n(%) | 22 (14.4) | 9 (47.4) | <0.001 |
| Diabetes history, yrs |  |  | 0.662 |
| <1 | 8 (5.2) | 1 (5.3) |  |
| 1-5 | 31 (20.3) | 3 (15.8) |  |
| 5-10 | 39 (25.5) | 8 (42.1) |  |
| 10-20 | 53 (34.6) | 5 (26.3) |  |
| >=20 | 22 (14.4) | 2 (10.5) |  |
| Preoperative DBP, mmHg | 74.26±8.80 | 77.42±15.19 | 0.385 |
| Glucose, mmol/L | 7.48±3.37 | 9.03±4.81 | 0.075 |
| Hemoglobin, g/L | 129.27±18.54 | 125±21.25 | 0.352 |
| Uric acid, umol/L | 325.28±103.86 | 367.89±127.01 | 0.102 |
| Baseline creatinine, umol/L | 82.08±48.19 | 79.42±50.47 | 0.822 |
| Albumin, g/L | 39.99±3.23 | 37.19±3.76 | 0.001 |
| Urine Protein level, n(%) |  |  | 0.025 |
| 0 | 131 (85.6) | 15 (78.9) |  |
| 1 | 16 (10.5) | 3 (15.8) |  |
| 2 | 6 (3.9) | 0 (0.0) |  |
| 3 | 0 (0.0) | 1 (5.3) |  |
| LVEF, % | 58.17±9.81 | 46.95±11.55 | <0.001 |
| Vessels of coronary artery disease, n(%) |  |  | 0.189 |
| 0 | 2 (1.3) | 1 (5.3) |  |
| 1 | 26 (17.0) | 3 (15.8) |  |
| 2 | 68 (44.4) | 5 (26.3) |  |
| 3 | 53 (34.6) | 8 (42.1) |  |
| 4 | 4 (2.6) | 2 (10.5) |  |
| Volume of contrast agent, mL | 135.29±69.27 | 121.05±63.06 | 0.395 |
| Diuretics, n(%) | 33 (21.6) | 15 (78.9) | <0.001 |

Abbreviations: CIAKI, contrast-induced acute kidney injury; CHF, congestive heart failure; ACS, acute coronary syndrome; DBP, diastolic blood pressure; LVEF, left ventricular ejection fraction.





**Supplementary Figure 1.** Ten-fold cross-validation results of AUC and accuracy in models.


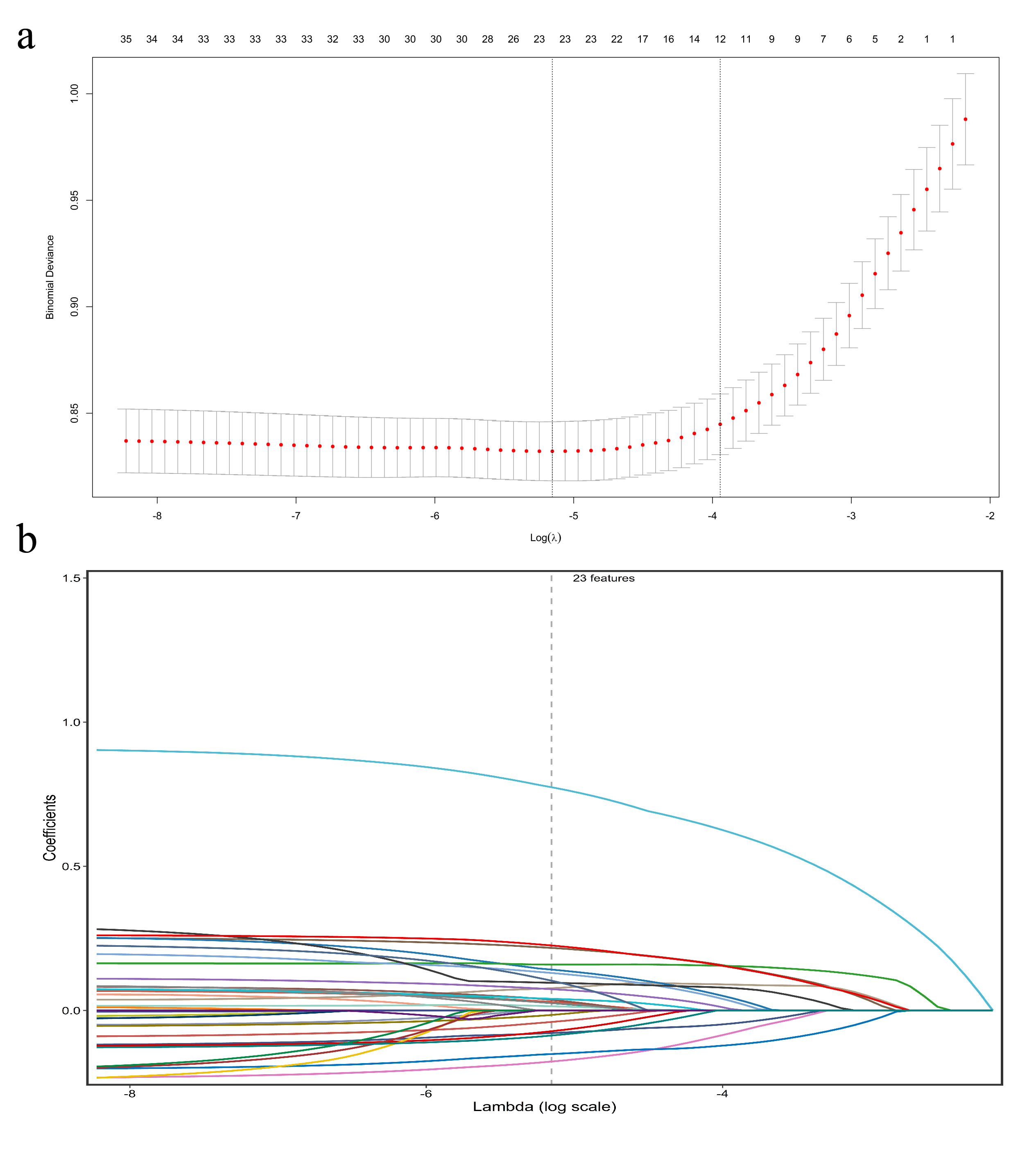


**Supplementary Figure 2.** Feature screening process of LASSO. (a) Lasso ten-fold cross-validation determines the number of important features according to the binomial deviation(λ=0.005774419, n=23). (b) The dynamic change in risk factors with the penalty coefficient; the vertical line indicates the optimal λ (n=23).


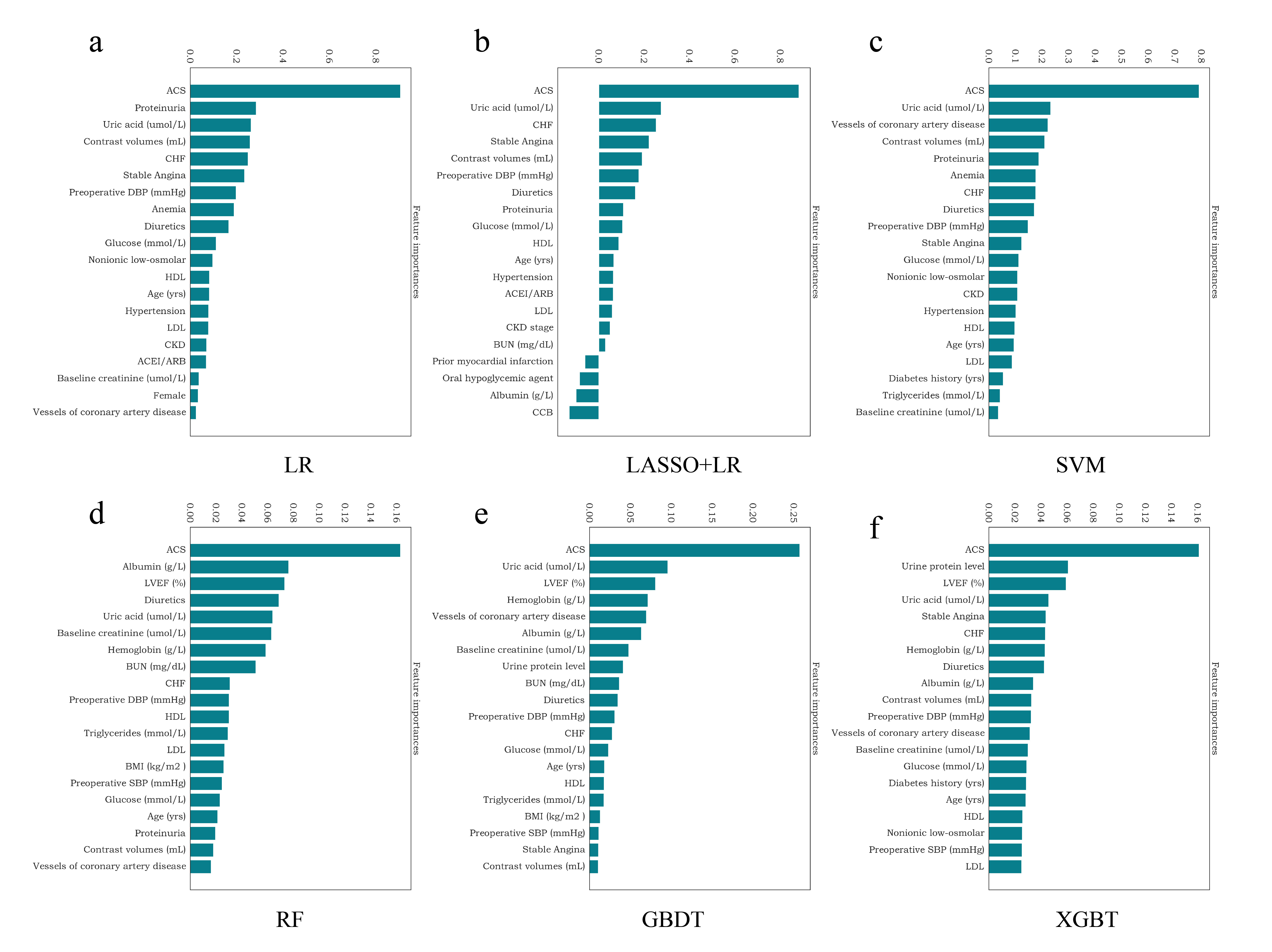


**Supplementary Figure 3.** The top 20 features of ML models.





**Supplementary Figure 4.** The prediction process of a patient on the dynamic explainable CIAKI risk calculator.
